# Strength of Wooden Truss Connections with Nail Plates Under Cyclic Humidity Changes

**DOI:** 10.3390/ma19163542

**Published:** 2026-08-21

**Authors:** Marek Wieruszewski, Adam Czerwiński, Agnieszka Katarzyna Wdowiak-Postulak, Maciej Jarzębski, Adrian Trociński

**Affiliations:** 1 Department of Mechanical Wood Technology, Poznań University of Life Sciences, 60-637 Poznań, Poland; marek.wieruszewski@up.poznan.pl (M.W.); czerwik4@gmail.com (A.C.);; 2 Department of Materials Strength and Structural Diagnostics, Kielce University of Technology, 25-314 Kielce, Poland; 3 Department of Physics and Biophysics, Faculty of Food Science and Nutrition, Poznań University of Life Sciences, Wojska Polskiego 38/42, 60-637 Poznań, Poland; maciej.jarzebski@up.poznan.pl

**Keywords:** metal-plate-connected joints, roof trusses, timber connections, moisture cycling, apparent joint stiffness, bending strength

## Abstract

**Highlights:**

- The mean apparent MPC stiffness decreased by approximately 12% across the test sequence, but the repeated-measures effect was not statistically significant.
- Including all 15 MPC specimens, the mean apparent bending strength was 16.92 MPa versus 34.14 MPa for the continuous solid wood references.
- Failure of MPC specimens progressed mainly through plate slip and partial spike withdrawal rather than abrupt timber fracture.
- Because no unexposed MPC control group was included, the observed stiffness change is interpreted as a combined conditioning-and-repeated-loading trend rather than a moisture-only effect.

**Abstract:**

Metal-plate-connected (MPC) joints govern the stiffness and load-bearing performance of many prefabricated timber roof trusses, yet their response to repeated moisture changes remains critical for serviceability and durability. This study evaluated five continuous C24 Norway spruce reference specimens and fifteen specimens joined with GNA20-MIT nail plates using sequential four-point-bending stiffness measurements and wetting–drying conditioning. Test I was used as the initial stiffness stage, whereas Tests II and III followed successive 24 h water-immersion and 6-day natural-drying intervals; the specimens were subsequently tested to failure. The mean apparent modulus of elasticity of the MPC specimens decreased from 1.39 to 1.22 GPa (approximately 12%), but a Friedman repeated-measures test did not show a statistically significant stage effect (χ^2^(2) = 4.13, *p* = 0.127). Because the same specimens were repeatedly loaded, and no unexposed MPC control group was included, this change cannot be attributed exclusively to moisture cycling. In the primary analysis retaining all 15 MPC specimens, the mean apparent bending strength of the connected elements was 16.92 MPa, compared with 34.14 MPa for the structurally different continuous reference specimens; excluding M7 yielded 17.87 MPa only as a sensitivity analysis. Failure of the connected specimens was progressive and dominated by plate slip and partial spike withdrawal, whereas solid specimens failed more abruptly in bending. The results therefore support attention to connection flexibility and serviceability under variable environmental and loading histories, while further controlled testing is required to isolate the specific contribution of moisture cycling.

## 1. Introduction

Roof trusses are primary load-bearing systems that transfer permanent actions and variable actions, including snow and wind, to the supports. Their design and execution directly affect structural safety and durability. In timber structures, connection behavior is often decisive for global stiffness and load-bearing capacity [1,2].

Metal connector plates, commonly called nail plates, are widely used in prefabricated timber roof trusses. They consist of thin steel sheets, typically 1–2 mm thick, with regularly arranged teeth that are pressed into the timber. The process produces repeatable and relatively stiff joints without separate nails or screws, making the system suitable for industrial prefabrication [3].

**Figure 1:**
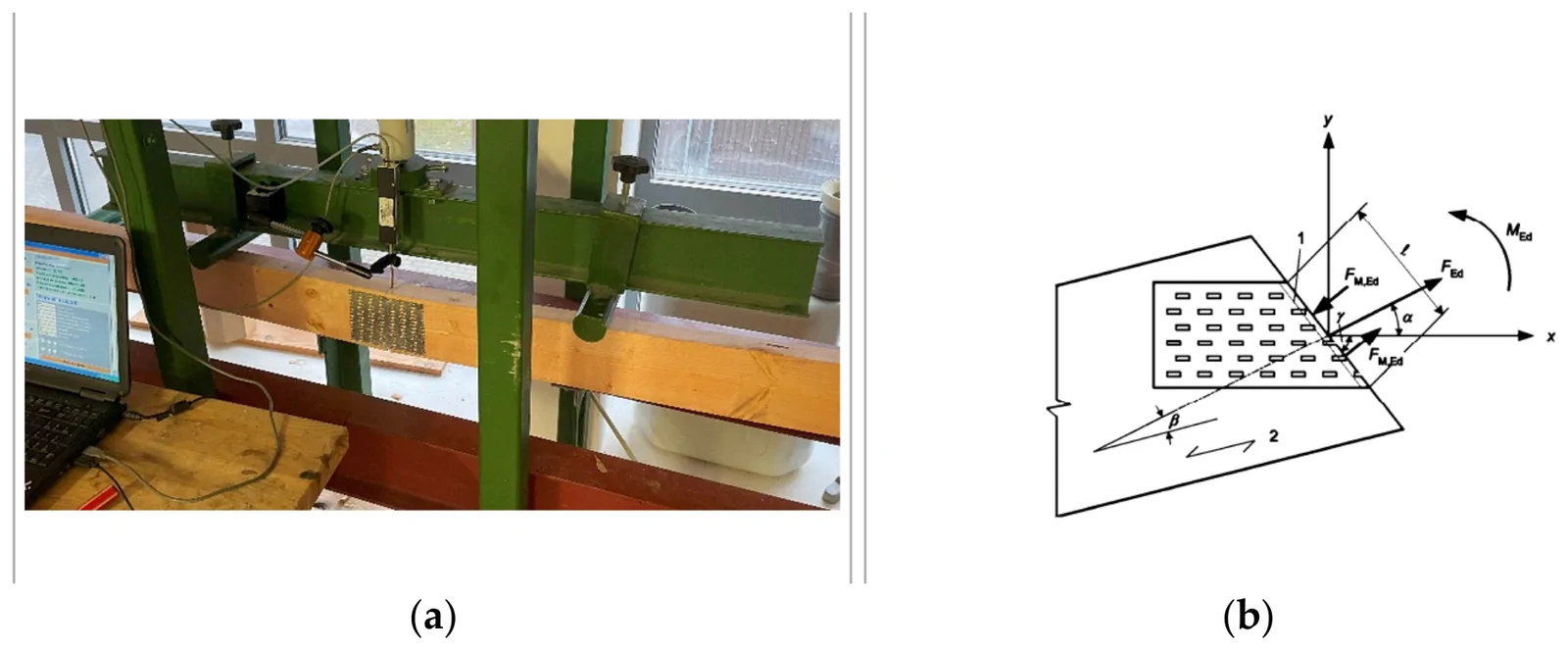
Structural context and mechanics of a metal-plate-connected (MPC) joint: (**a**) laboratory specimen showing the central joint and connector in the four-point bending apparatus; (**b**) definition of the plate axes, grain direction, force components, moment, and angles used in anchorage and plate resistance calculations. The node is structurally important because local slip or loss of tooth anchorage changes joint rotation, truss deflection, and internal-force redistribution. Panel (**b**) adapted from Eurocode 5 [4]; photograph from the experimental campaign.

Figure 1 places the tested connector within the structural hierarchy of a complete prefabricated roof truss. Because axial forces from the chords and web members converge at these semi-rigid nodes, local plate slip, tooth withdrawal, or moisture-induced loss of anchorage can alter both the stiffness of the joint and the distribution of forces in the entire truss.

Metal-plate-connected truss technology developed rapidly with the growth of industrial timber prefabrication. Contemporary design procedures combine structural analysis of the truss with verification of timber members, plate anchorage, and plate resistance in accordance with Eurocode 5 and dedicated truss standards [4,5].

In the European market, system suppliers provide both connector plates and dedicated design software. These tools enable structural modeling, verification of ultimate and serviceability limit states, and preparation of production documentation. The joint model must nevertheless reflect plate orientation, effective anchorage area, and the semi-rigid response of the wood–steel interface [4,5,6].

The mechanical response of MPC joints depends on connector geometry, joint scale, loading direction, and loading history. Experimental and numerical studies have shown that these parameters influence stiffness, slip, force redistribution, and failure mode [6,7,8,9].

The load-bearing capacity and stiffness of nail-plate connections depend on timber density and moisture content, the presence of defects, the dimensions and orientation of the plate, and the quality of pressing. These variables govern tooth anchorage, local crushing, shear transfer, and tensile resistance in the connection zone [1,3,6].

Moisture is a particularly important environmental factor because wood is hygroscopic. Repeated swelling and shrinkage can relax the wood fibers around the plate teeth, increase joint slip, and reduce initial stiffness. Moisture-cycling and soaking studies have demonstrated progressive deterioration of MPC joint response under such conditions [10,11].

Design standards account for environmental and load-duration effects through service classes and modification/deformation factors. Eurocode 5 and ANSI/TPI 1-2022 require the designer to consider the expected service environment, load duration, material properties, and connector resistance when verifying timber trusses [4,5]. The present accelerated wetting–drying history should not, however, be treated as a direct experimental calibration of Eurocode 5 modification factors.

Accordingly, the environmental durability of nail plate connections is relevant to both ultimate and serviceability limit states. Previous studies indicate that cyclic wetting and drying can influence connection stiffness and permanent deformation and, where corrosion protection is insufficient, may also affect the steel connector [9,11]. In this study, corrosion was not measured and is therefore discussed only as a limitation for long-term extrapolation.

The aim of this study is to quantify changes in the apparent stiffness and final load-bearing response of spruce members containing metal-plate-connected butt joints during a laboratory sequence that combines repeated loading with cyclic wetting and drying. The work compares connected specimens with continuous solid wood references and documents the observed failure mechanisms. Because the program did not include an unexposed MPC control group or local displacement measurements at the joint, the study does not attempt to separate moisture effects from repeated loading, plate seating, or joint slip effects.

Central research question. Do sequential wetting–drying conditioning and repeated stiffness measurements coincide with a measurable change in the apparent flexural stiffness of MPC specimens, and what failure mechanisms govern the final connected-element response?

## 2. Materials and Methods

### 2.1. Material

The material consisted of four-sided, planed, kiln-dried Norway spruce (*Picea abies*) structural timber assigned to strength class C24 (Table 1). Selection followed the characteristic strength, stiffness, and density requirements of EN 338 [12]. The timber originated from a single production batch sourced from Långasjö, Sweden, which limited variation associated with origin, drying history, and processing. Twenty specimens were prepared: fifteen specimens containing a simple butt joint connected by nail plates and five continuous solid wood reference specimens. The continuous reference series provides a structural material benchmark, but the small sample size (n = 5) and different geometries of the series components mean that it cannot isolate the specific effect of moisture cycling on MPC joints.

### 2.2. Sample Preparation

**Figure 2:**
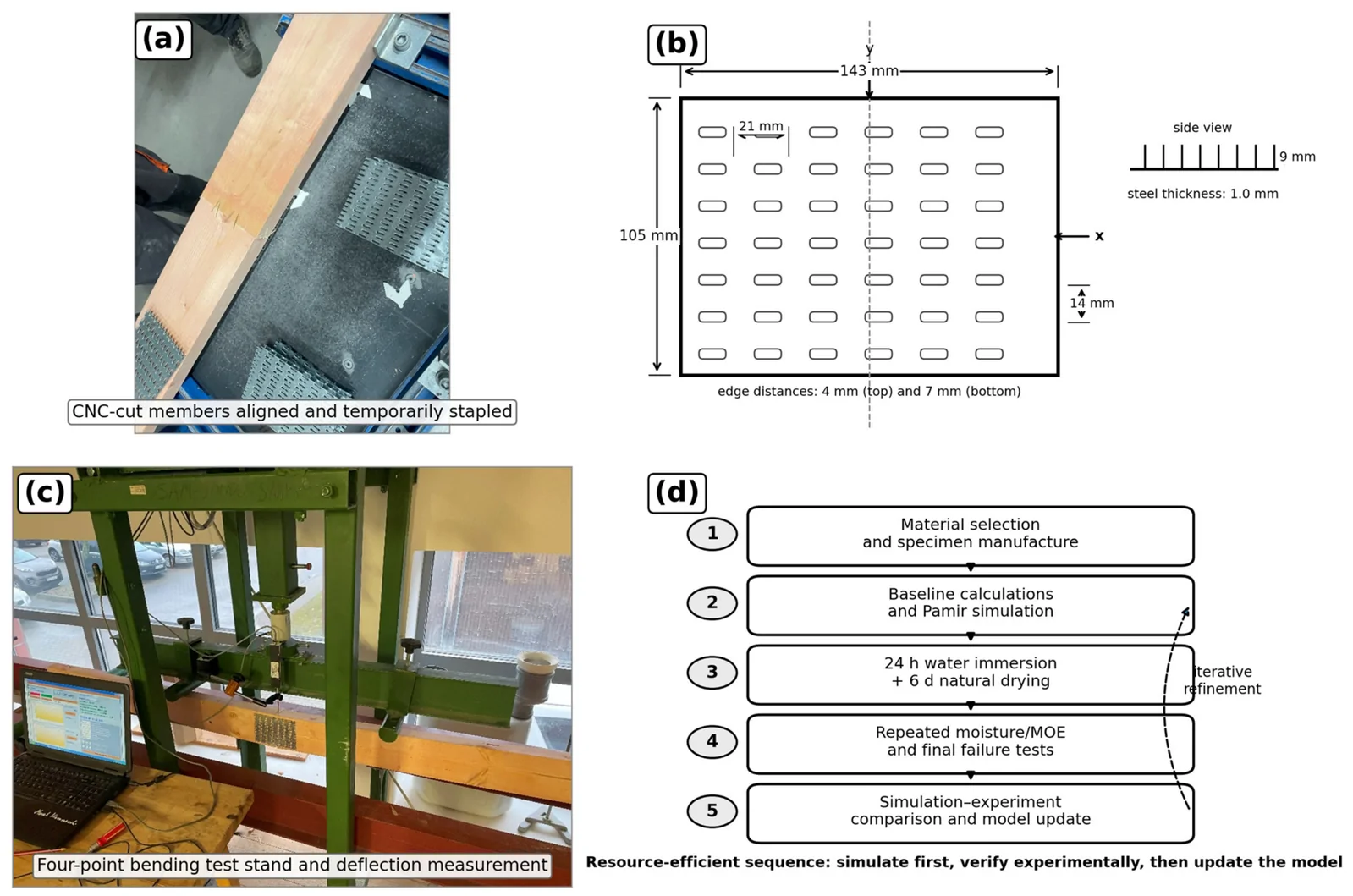
Experimental preparation and methodological workflow: (**a**) CNC-cut spruce members aligned on a magnetic table and temporarily stapled across the central butt joint before plate pressing; (**b**) GNA20-MIT connector geometry, including the 143 × 105 mm plate dimensions, 1.0 mm steel thickness, 9 mm tooth height, 21 mm longitudinal pitch, 14 mm transverse pitch, and indicated edge distances; (**c**) four-point bending test stand with the central joint positioned in the constant-moment region and instrumentation used to record load and global deflection; (**d**) clarified experimental sequence showing the initial stiffness stage (Test I), successive wetting–drying intervals leading to Tests II and III, the final destructive test, and comparison with the computational model. Photographs from the experimental campaign; diagrams are the authors’ own work.

All elements were cut on a Homag CNC machining apparatus with a dimensional tolerance of 1 mm. Each specimen had an overall length of 1920 mm and a nominal cross-section of 45 × 120 mm. The connected specimens were assembled as central, end-to-end butt joints representative of simplified chord connections in prefabricated roof trusses. MiTek GNA20-MIT steel nail plates measuring 105 × 143 × 1.0 mm were applied symmetrically on both faces. The members were aligned on magnetic tables, temporarily stapled to prevent displacement, and pressed using a MARK 3 hydraulic press at approximately 18 t. Plate centering, full tooth insertion, member alignment, and the width of the butt joint gap were controlled, because inaccurate seating changes the effective anchorage area, initial slip, and force transfer [5,13]. The fabrication sequence, connector geometry, and four-point bending test stand are shown in Figure 2.

#### Specimen Groups, Nomenclature, and Geometry

The letter A identifies the continuous solid wood reference series, whereas M identifies specimens containing the central metal-plate-connected joint. The numerical suffix denotes the individual specimen within each series. All samples were measured for density and moisture content, subjected to repeated stiffness measurements, and finally tested to failure in four-point bending; the connected series additionally enabled direct observation of plate slip and tooth withdrawal mechanisms (Table 2).

### 2.3. Methods

The research program comprised five stages: (i) material selection and specimen manufacture; (ii) baseline manual calculations and Pamir simulation; (iii) initial and repeated stiffness measurements combined with cyclic moisture conditioning; (iv) final destructive testing and measurement of physical properties; and (v) comparison of the experimental response with the computational model. The workflow is summarized in Figure 2d. It is used here as an organizational sequence rather than as evidence of formal model updating or quantified environmental benefit.

#### 2.3.1. Computational Analysis

A parallel computational analysis was performed in Pamir (PAMIR Viewer, Pamir Project—Mitek Industries Polska), a software program used for the design of prefabricated timber roof structures with nail plate connections. The model reproduced the specimen geometry, support conditions, and laboratory loading. It was used to verify internal forces, timber member utilization, plate anchorage, and plate resistance according to Eurocode 5 and EN 14545 [4,13]. The computational stage preceded destructive testing so that critical force paths, connector utilization, and expected failure regions could be identified before experimental verification.

The strength characteristics of the nail plate were adopted from the manufacturer’s declared performance and the procedures for timber connectors. The analysis included anchorage resistance in timber; tensile, compressive, and shear resistance of the plate; and plate orientation relative to the grain (Table 3). The x- and y-axes and the angles α, β, and γ were defined in accordance with the geometry used in Eurocode 5 and EN 14545 [4,13]. The adopted coordinate system and force angle definitions are illustrated in Figure 1b.

#### 2.3.2. Load-Bearing Capacity of the Nail Plate Anchorage

The load-bearing capacity of the plate anchorage should be determined on the basis of tests or formulas:

$${ƒ}_{a,\alpha ,\beta ,k}=\;max\left\{\begin{array}{c} {ƒ}_{a,\alpha ,0,k}-\left({ƒ}_{a,\alpha ,0,k}-\;{ƒ}_{a,90,90,k}\right)\frac{\beta }{45{}^{\circ}}\\ {ƒ}_{a,0,0,k}-\left({ƒ}_{a,0,0,k}-\;{ƒ}_{a,90,90,k}\right)\sin(\max\left(\alpha ,\beta )\right) \end{array}\right.\;\;gdy\;\beta \leq 45{}^{\circ}$$

$${ƒ}_{a,\alpha ,0,k}={ƒ}_{a,0,0,k}-\left({ƒ}_{a,0,0,k}-{ƒ}_{a,90,90,k}\right)\sin(\max(\alpha ,\beta ))\;\;\;\;\;\;\;gdy\;45{}^{\circ}<\beta \leq 90{}^{\circ}$$

$$f_{a,\alpha ,0,k}=f_{a,0,0,k}+k_{1}\alpha \;\mathrm{When}\;\alpha \leq \alpha_{0}$$

$$f_{a,\alpha ,0,k}=f_{a,0,0,k}+k_{1}\alpha_{0}+k_{2}(\alpha -\alpha_{0})\;\mathrm{When}\;\alpha_{0}<\alpha \leq 90{}^{\circ}$$

The forces acting in the joint are determined from the formula

$$F_{A,Ed}=\frac{F_{Ed}}{2}$$

or when the average distance between the connected elements is less than 1.5 mm, and the maximum width does not exceed 3 mm. Then, for the calculations we can adopt the following value:

$$F_{A,Ed}=\frac{F_{Ed}}{4}$$

In the connection of elements compressed along the length, the pressure is taken into account using the following formulas:

$$F_{A,Ed}=\sqrt{{\left(\frac{F_{Ed}\cos\beta }{2}-\frac{3\left|M_{Ed}\right|}{2h}\right)}^{2}+{\left(F_{Ed}\sin\beta \right)}^{2}\;}$$

$$M_{A,Ed}=\frac{M_{Ed}}{2}$$

where

$F_{A,Ed}$—calculated force applied at the center of gravity of the effective surface of the plate

$M_{A,Ed}$—design moment applied at the center of gravity of the effective surface of the plate

$F_{Ed}$—calculated axial force in the belt

h—waist height

Geometric parameters are determined from the formulas

$$d=\sqrt{{\left(\frac{A_{ef}}{h_{ef}}\right)}^{2}+h_{ef}^{2}}$$

$$W_{p}=\frac{A_{ef}d}{4}$$

where

$W_{p}$—plate strength index

$A_{af}$—effective surface: the contact surface area of the plate with the element, reduced by 5 mm at the side edges of the element and by a dimension equal to six nominal connector thicknesses, counted along the grain from the end of the element

$h_{ef}$—maximum height of the effective anchorage surface, perpendicular to the largest dimension of the plate

The load-bearing capacity of the plate anchorage meets the following condition:

$$\tau_{F,d}=\frac{F_{A,Ed}}{A_{ef}}$$

$$\tau_{M,d}=\frac{M_{A,Ed}}{W_{p}}$$

$${\left(\frac{\tau_{F,d}}{\frac{f_{a,\alpha ,\beta ,k}}{\gamma_{M}}}\right)}^{2}+{\left(\frac{\tau_{M,d}}{\frac{f_{a,0,0,k}}{\gamma_{M}}}\right)}^{2}\leq 1$$

$\gamma_{M}=1.3$ according to the National Annex

#### 2.3.3. Load-Bearing Capacity of the Nail Plate

The load-bearing capacity of the plate was determined based on the formulas

$$F_{x,Rk}=\max\left\{\begin{array}{c} |f_{n,0,k}l\cdot \sin(\gamma -\gamma_{0}\sin2\gamma )|\\ \left|f_{v,0,k}l\cdot \cos\gamma \right| \end{array}\right.$$

$$F_{y,Rk}=\max\left\{\begin{array}{c} \left|f_{n,90,k}l\cdot \cos\gamma \right|\\ {|kf}_{v,90,k}l\cdot \sin\gamma | \end{array}\right.$$

$$f_{n,0,k}=\left\{\begin{array}{c} f_{t,0,k}\;dla\;F_{x,Ed}>0\\ f_{c,0,k}\;dla\;F_{x,Ed}<0 \end{array}\right.$$

$$f_{n,90,k}=\left\{\begin{array}{c} f_{t,90,k}\;dla\;F_{x,Ed}>0\\ f_{c,90,k}\;dla\;F_{x,Ed}<0 \end{array}\right.$$

$$k=\left\{\begin{array}{c} 1+k_{v}\sin\left(2\gamma \right)dlaF_{x,Ed}>0\\ 1\;dla\;F_{x,Ed}<0 \end{array}\right.$$

where

$k_{v},\;\gamma_{0}$—constants determined on the basis of tests (provided by the manufacturer)

The forces acting in the joint in the two main directions of the plate are determined from the formulas

$$F_{x,Ed}=F_{Ed}\cos\alpha \pm 2F_{M,Ed}\sin\gamma$$

$$F_{y,Ed}=F_{Ed}\sin\alpha \pm 2F_{M,Ed}\cos\gamma$$

$F_{Ed}$—design force in the plate (equal to half the force in the element)

$$F_{M,Ed}=\frac{2M_{Ed}}{l}$$

The load-bearing capacity of the plate must meet the following condition:

$${\left(\frac{F_{x,Ed}}{\frac{F_{x,Rk}}{\gamma_{M}}}\right)}^{2}+{\left(\frac{F_{y,Ed}}{\frac{F_{y,Rk}}{\gamma_{M}}}\right)}^{2}\leq 1$$

Table 4 presents the performance characteristics declared by the manufacturer for the GNA20-MIT nail plate used in this study [14].

#### 2.3.4. Determination of Wood Density

Wood density was determined using the stereometric method. Before the strength tests were performed, samples were weighed to the nearest 0.1 g, width and thickness were measured using a caliper with an accuracy of 0.05 mm, and length was measured using a 0–5 m tape measure with an accuracy of 0.1 mm. Sample density was calculated according to the following equation:

$$\rho =\frac{m}{v}$$

where

ρ—density [kg/m^3^]

m—weight [kg]

v—volume [m^3^]

#### 2.3.5. Determination of the Bending Modulus of Elasticity

The modulus of elasticity was determined in four-point static bending in accordance with EN 408 [15]. Load was increased within the proportional range, and deflection was measured using a dial gauge with a resolution of 0.01 mm. The support span was 16 h, and the two loading points were arranged symmetrically. The initial seating load of 50 N was excluded from the calculation.

The modulus of elasticity during bending was determined based on the formula

$$E_{w}=\frac{a\cdot l^{2}\cdot \Delta F}{4bh^{3}\cdot \Delta f}$$

where

*Ew*—value of the modulus of elasticity along the fibers of laminated wood,

when bending in a plane perpendicular to the adhesive joints [Pa],

*a*—distance from the place where the loading force is applied to the nearest support [m],

*l*—support spacing [m],

*ΔF*—load increase in a given measurement range [N],

*Δf*—deflection arrow increase in a given measurement range [m],

*B*—width of the cross-section of the sample,

*h*—height of the cross-section of the sample.

The measured modulus of elasticity at the actual moisture content W was converted to the reference moisture content of 12% using the relationship specified in PN-63/D-04117 [16]:

$$E=E_{w}\left(1+\alpha \left(w-12\right)\right)$$

where

*E_W_*—modulus of elasticity at actual humidity during the “W” tests [N/mm^2^],

*α*—conversion factor, α = 0.04 (in accordance with PN-63/D-04117),

*W*—absolute humidity of the sample [%].

**Figure 3:**
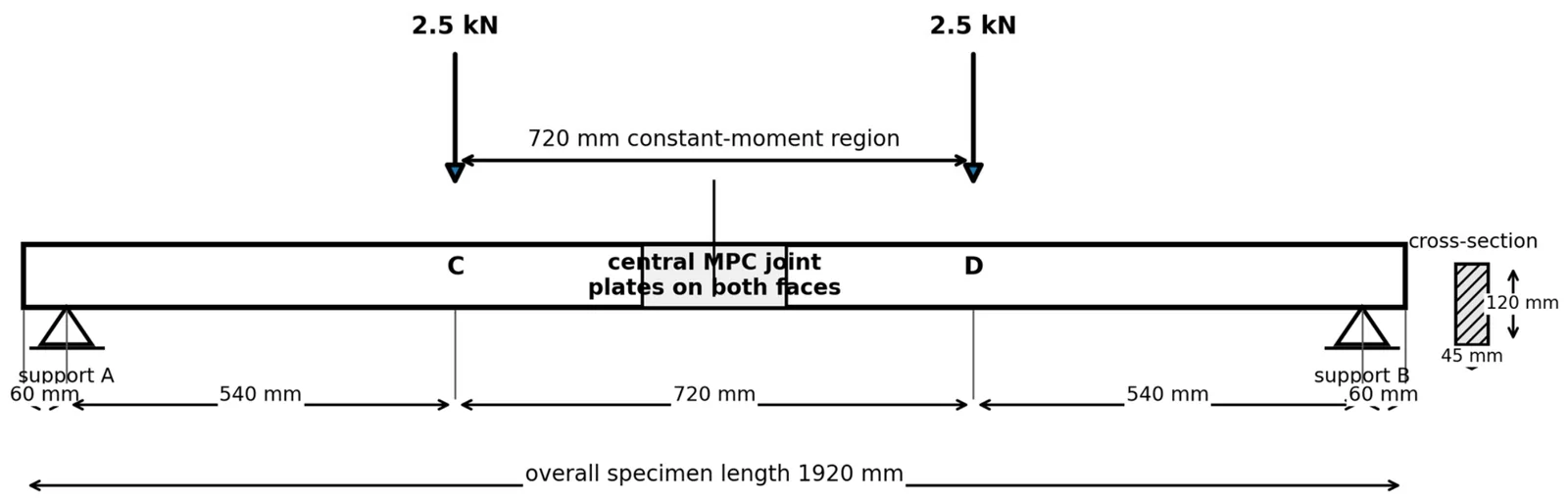
Four-point bending configuration used for stiffness and ultimate load testing. Supports A and B were positioned 60 mm from the specimen ends. The two loading heads acted at points C and D, producing 540 mm outer shear spans and a 720 mm constant-moment region. The drawing shows the nominal 2.5 kN load at each loading point used to define the test scheme; during the strength test, the load was increased monotonically to failure. The 1920 mm long specimen had a 45 × 120 mm cross-section, and the MPC joint was centered between C and D so that connector deformation governed the measured joint response (own drawing).

The specimen geometry and loading arrangement presented in Figure 3 were used in the experimental procedure and calculations.

#### 2.3.6. Determination of Bending Strength and Ultimate Load

Bending strength and ultimate load were determined by monotonically increasing the four-point bending load until failure. Bending strength was calculated in accordance with EN 408 [15] and converted to the 12% reference moisture content using PN-77/D-04103 and PN-EN 384 [17,18]:

$$f_{m}=\frac{aF_{max}}{2W}$$

$$E_{w}=\frac{a\cdot l^{2}\cdot \Delta F}{4bh^{3}\cdot \Delta f}$$

where

*f_m_*—bending strength [Pa],

*a*—distance from the place where the loading force is applied to the nearest support [m],

*F_max_*—breaking load [N],

*W*—section strength modulus [m^3^]

The value of the beam’s bending strength at the time of testing was converted to the value at 12% humidity using the following formula (PN-EN 384):

$$f_{12}=f_{m}\left(1+\alpha \left(w-12\right)\right)$$

where

*f*_12_—bending strength at reference humidity w = 12%,

*f_m_*—bending strength determined during testing,

*α*—coefficient of change in wood strength when its moisture content changes by 1%, which for spruce is 0.04 (PN-77/D-04103).

#### 2.3.7. Cyclic Humidification and Drying Program

The accelerated ageing procedure was adapted from PN-EN 84 and PN-EN 73 [19,20]. After the seating/preload step used to stabilize the setup, Test I served as the initial stiffness measurement. The interval from Test I to Test II comprised 24 h of full water immersion at 4–15 °C followed by 6 days of drying under laboratory conditions (20 °C and 40% relative humidity); the same wetting–drying interval was then repeated before Test III. Moisture content was measured with an electrical resistance meter in accordance with PN-EN 13183-2 [21], and four-point bending within the proportional range was used to determine the apparent modulus of elasticity at each test stage. After Test III, the specimens were loaded monotonically to failure.

During Tests I–III, moisture content and elastic deflection were recorded for stiffness determination. Ultimate failure force and apparent bending strength were obtained only during the final destructive test after Test III; therefore, the present dataset does not provide cycle-by-cycle ultimate capacity degradation. Results were compiled separately for continuous reference specimens and MPC specimens using descriptive statistics.

#### 2.3.8. Statistical Analysis

Because the same 15 MPC specimens were measured at Tests I–III, stage-to-stage changes in apparent modulus were evaluated using the Friedman repeated-measures test. Exploratory pairwise comparisons used the Wilcoxon signed-rank test. For the final apparent bending-strength series, a two-sided Grubbs test at α = 0.05 was used to identify a statistically unusual observation. Regardless of the Grubbs result, all specimens were retained in the primary engineering analysis; exclusion of M7 was used only as a sensitivity analysis. The five continuous reference specimens were treated descriptively because of the small sample size and the pronounced instability of their repeated apparent modulus values.

## 3. Results

The results are presented from computational verification through physical properties, repeated apparent stiffness measurements, final apparent bending strength, and observed failure mechanisms. The interpretation distinguishes descriptive stage-to-stage changes from causal effects: the present design permits characterization of the combined conditioning/repeated-loading sequence, but it does not isolate moisture cycling as a single factor.

### 3.1. Numerical Analysis and Model Verification

**Figure 4:**
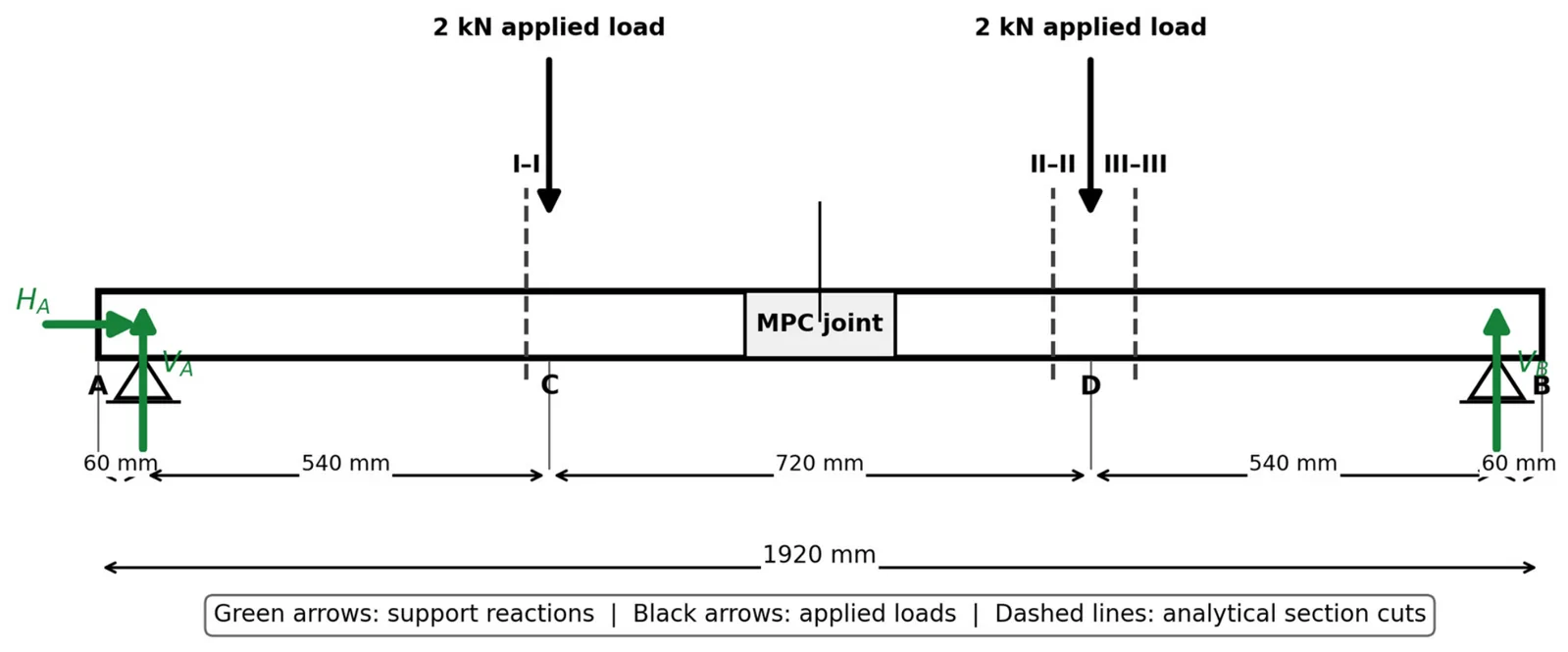
Static scheme adopted for the manual internal force analysis. Supports are located at A and B, while two concentrated loads of 2 kN act at C and D. Thick green arrows represent the reactions H_A, V_A, and V_B; black arrows represent the applied loads. Dashed lines I–I, II–II, and III–III divide the beam into the intervals used to derive the normal force N(x), shear force T(x), and bending moment M(x) functions. The central shaded field marks the MPC joint (authors’ own drawing).

**Figure 5:**
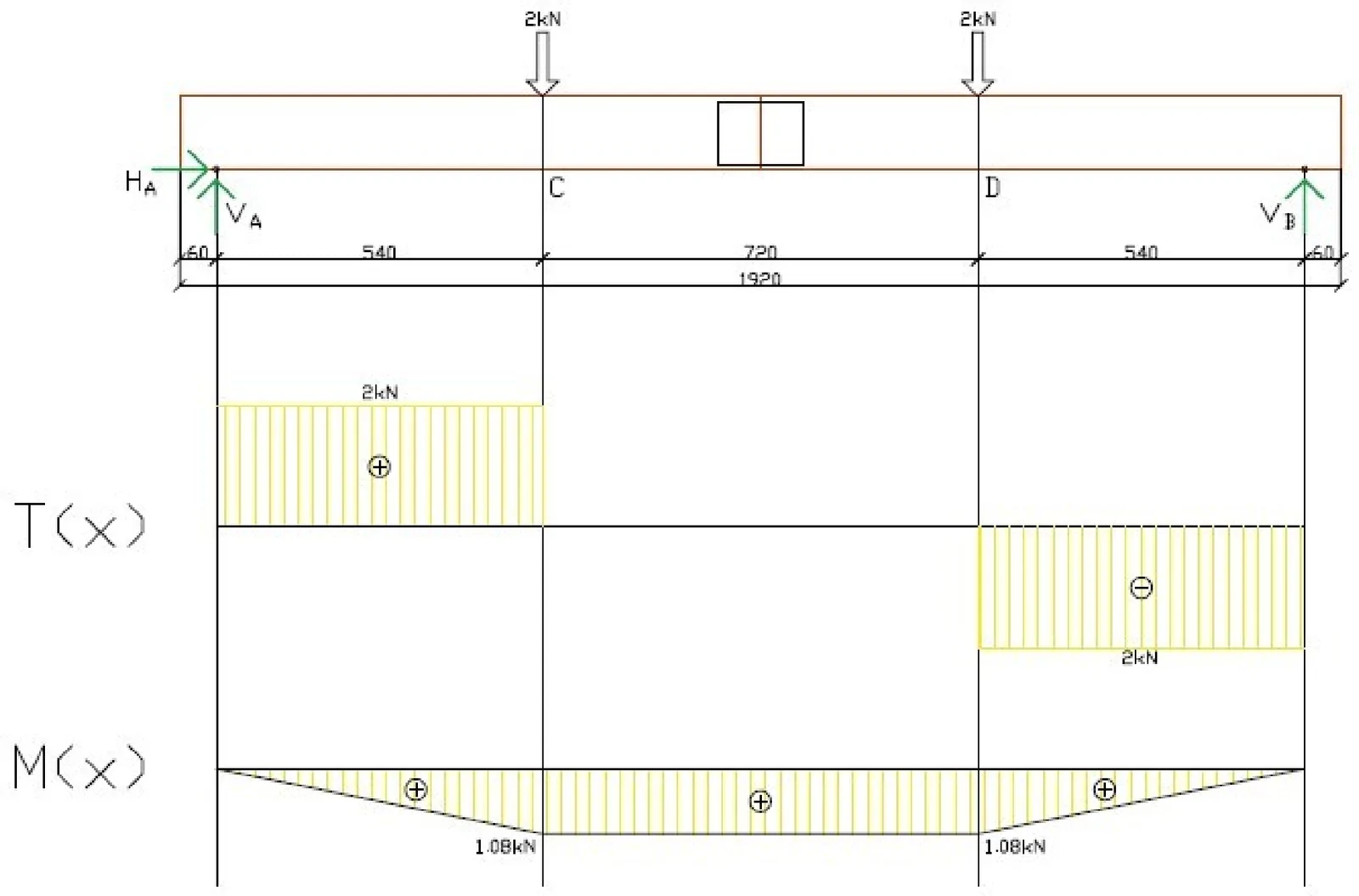
Shear force T(x) and bending moment M(x) diagrams for the analyzed four-point bending system. The shear force is +2 kN in the left outer span, 0 between the loading points, and −2 kN in the right outer span; the bending moment reaches a constant maximum of approximately 1.08 kN·m in the central region containing the joint (own drawing).

To determine the internal forces in the bent specimen, the support reactions and the piecewise functions N(x), T(x), and M(x) were derived from the static scheme in Figure 4 and summarized in Figure 5. In Figure 4, the thick green arrows denote the horizontal and vertical support reactions (H_A, V_A, and V_B), the black downward arrows denote the two applied 2 kN loads, and the dashed vertical lines identify the section cuts used in the analytical solution.

Determination of internal forces:

$$\sum Y=0;\;V_{A}+\;V_{B}-2\;kN-2\;kN=0$$

$$V_{B}=4\;kN-V_{A}$$

$$\sum M_{A}=0;\;2\;kN\cdot 0.54\;m+2\;kN\cdot \left(1.8\;m-0.54\;m\right)-1.8\;m\cdot V_{B}=0$$

$$V_{B}=\frac{1.08\;kN+2.52\;kN}{1.8\;m}=2\;kN$$

$$V_{A}=2\;kN$$

NTM Charts

Cutting of AC beam section II; $x\in \left\langle 0;0.54\;m)\right.$

$$N\left(x\right)={-H}_{A}=0\;kN$$

$$T\left(x\right)=V_{A}=2\;kN$$

$$M\left(x\right)=V_{A}\cdot x$$

$$M\left(0\right)=2\;kN\cdot 0\;m=0\;kN$$

$$M\left(0.54\right)=2\;kN\cdot 0.54\;m=1.08\;kN$$

Cutting of CD beam section II-II; $x\in \left\langle 0.54;1.26\;m)\right.$

$$N\left(x\right)={-H}_{A}=0\;kN$$

$$T\left(x\right)=V_{A}-2\;kN=0\;kN$$

$$M\left(x\right)=V_{A}\cdot x-2\;kN\cdot (x-0.54\;m)$$

$$M\left(0.54\right)=2\;kN\cdot 0.54\;m-2\;kN\cdot \left(0.54\;m-0.54\;m\right)=1.08\;kN$$

$$M\left(1.26\right)=2\;kN\cdot 1.26\;m-2\;kN\cdot \left(1.26\;m-0.54\;m\right)=1.08\;kN$$

Cutting of BD beam section III-III; $x\in \left\langle 0;0.54\;m)\right.$

$$N\left(x\right)=0\;kN$$

$$T\left(x\right)=-V_{B}=-2\;kN$$

$$M\left(x\right)=V_{A}\cdot x$$

$$M\left(0\right)=2\;kN\cdot 0\;m=0\;kN$$

$$M\left(0.54\right)=2\;kN\cdot 0.54\;m=1.08\;kN$$

### 3.2. Design Load-Bearing Capacity of the Timber Element

In order to check the design strength of the timber cross-section, the ultimate limit state method described in Eurocode 5 [4] was used (Table 1).

According to the internal force diagram, only shear forces and bending moments occur in the beam. Therefore, the bending and shear load-bearing capacity of the timber cross-section was checked.

#### 3.2.1. Bending Capacity

The load-bearing capacity of a wooden element for unidirectional bending is determined from the formula

$$\frac{\sigma_{m,y,d}}{f_{m,y,d}}\leq 1,$$

$\sigma_{m,y,d}$—design bending stresses

$f_{m,y,d}$—design bending strength

The bending stress is determined using the formula

$$\sigma_{m,y,d}=\frac{M_{y,d}}{W_{y}}$$

$M_{y,d}=1.08\;kNm$—according to the internal forces diagram

$W_{y}=$ strength index of a wooden cross-section

$$W_{y}=\frac{{b\cdot h}^{2}}{6}$$

$$W_{y}=\frac{{4.5\cdot 12}^{2}}{6}=108\;cm^{3}$$

$$\sigma_{m,y,d}=\frac{1080}{0.000108}=10000000\;\frac{N}{m^{2}}=10\;MPa$$

The design bending strength is determined from the formula

$$f_{m,d}=\frac{f_{m,k}k_{mod}k_{h}}{\gamma_{M}}\;,$$

$f_{m,k}=24\;MPa$—characteristic bending strength for C24 wood

$k_{mod}=0.9$—safety factor due to the duration of the load; the load was assumed to be short-term

$\gamma_{M}=1.3$—material property safety factor

$k_{h}$—coefficient due to the height of the element below 150 mm, determined from the formula

$$k_{h}=\min\left\{\begin{array}{c} {\left(\frac{150}{h}\right)}^{0.2}\\ 1.3 \end{array}\right.$$

h—height of the bending element [mm]

$$k_{h}=\min\left\{\begin{array}{c} {\left(\frac{150}{120}\right)}^{0.2}\\ 1.3 \end{array}=1.046\right.$$

$$f_{m,d}=\frac{24\cdot 0.9\cdot 1.046}{1.3}=17.38\;MPa$$

$$\frac{\sigma_{m,y,d}}{f_{m,y,d}}=\frac{10}{17.38}=0.575\leq 1$$

The bending load-bearing capacity of the wooden cross-section has been met.

#### 3.2.2. Shear Capacity

The shear capacity of a wooden element is determined from the formula

$$\frac{\tau_{d}}{f_{v,d}}\leq 1,$$

$\tau_{d}$—design shear stress

$f_{v,d}$—design shear strength

The shear stress for a rectangular cross-section can be determined from the formula

$$\tau_{d}=\frac{3T}{2A_{ef}}$$

T=2.0 kN—maximum shear force, determined according to the internal forces diagram

$A_{ef}=$ effective cross-sectional area of wood

When checking the shear resistance of bending elements, the influence of cracks should be taken into account by introducing the effective width of the element:

$$b_{ef}=k_{cr}b$$

$k_{cr}=0.67$ for solid wood

$$b_{ef}=0.67\cdot 4.5=3.015\;cm$$

$$A_{ef}=b_{ef}\cdot h$$

$$A_{ef}=3.015\cdot 12=36.18\;{cm}^{2}$$

$$\tau_{d}=\frac{3\cdot 2000}{2\cdot 0.003618}=829187\;\frac{N}{m^{2}}=0.829\;mPa$$

The design shear strength is determined from the formula

$$f_{m,d}=f_{v,d}=\frac{f_{v,k}k_{mod}}{\gamma_{M}},\;$$

$f_{v,k}=4\;MPa$—characteristic shear strength for C24 wood

$k_{mod}=0.9$—safety factor due to the load duration; the load was assumed to be short-term

$\gamma_{M}=1.3$—material property safety factor

$$f_{v,d}=\frac{4\cdot 0.9}{1.3}=2.769\;MPa$$

$$\frac{\tau_{d}}{f_{v,d}}=\leq 1\frac{0.829}{2.769}=0.30\leq 1$$

The shear capacity of the timber section has been met.

### 3.3. Load-Bearing Capacity of the Nail Plate Connection

The load-bearing capacity of the nail plate connection was checked under the action of a force of 4.0 kN.

#### 3.3.1. Anchorage Capacity

Determination of the characteristic load-bearing capacity of the plate anchorage along the grain:

α=0°

$$f_{a,\alpha ,0,k}=f_{a,0,0,k}+k_{1}\alpha$$

$$f_{a,\alpha ,0,k}=2.83$$

Determination of the characteristic load-bearing capacity of the plate anchorage:

$$f_{a,\alpha ,\beta ,k}=max\left\{\begin{array}{c} f_{a,\alpha ,0,k}-(f_{a,\alpha ,0,k}-f_{a,90,90,k})\frac{\beta }{45{}^{\circ}}\;\\ f_{a,0,0,k}-(f_{a,0,0,k}-f_{a,90,90,k})sin(\max\left(\alpha ,\beta )\right) \end{array}\right.\mathrm{When}\;\beta \leq 45{}^{\circ}$$

$$f_{a,\alpha ,\beta ,k}=max\left\{\begin{array}{c} 2.83-\left(2.83-1.63\right)\frac{0{}^{\circ}}{45{}^{\circ}}=2.83\\ 2.83-\left(2.83-1.63\right)\sin0{}^{\circ}=2.83 \end{array}=2.83\;\frac{N}{mm^{2}}\right.$$

**Figure 6:**
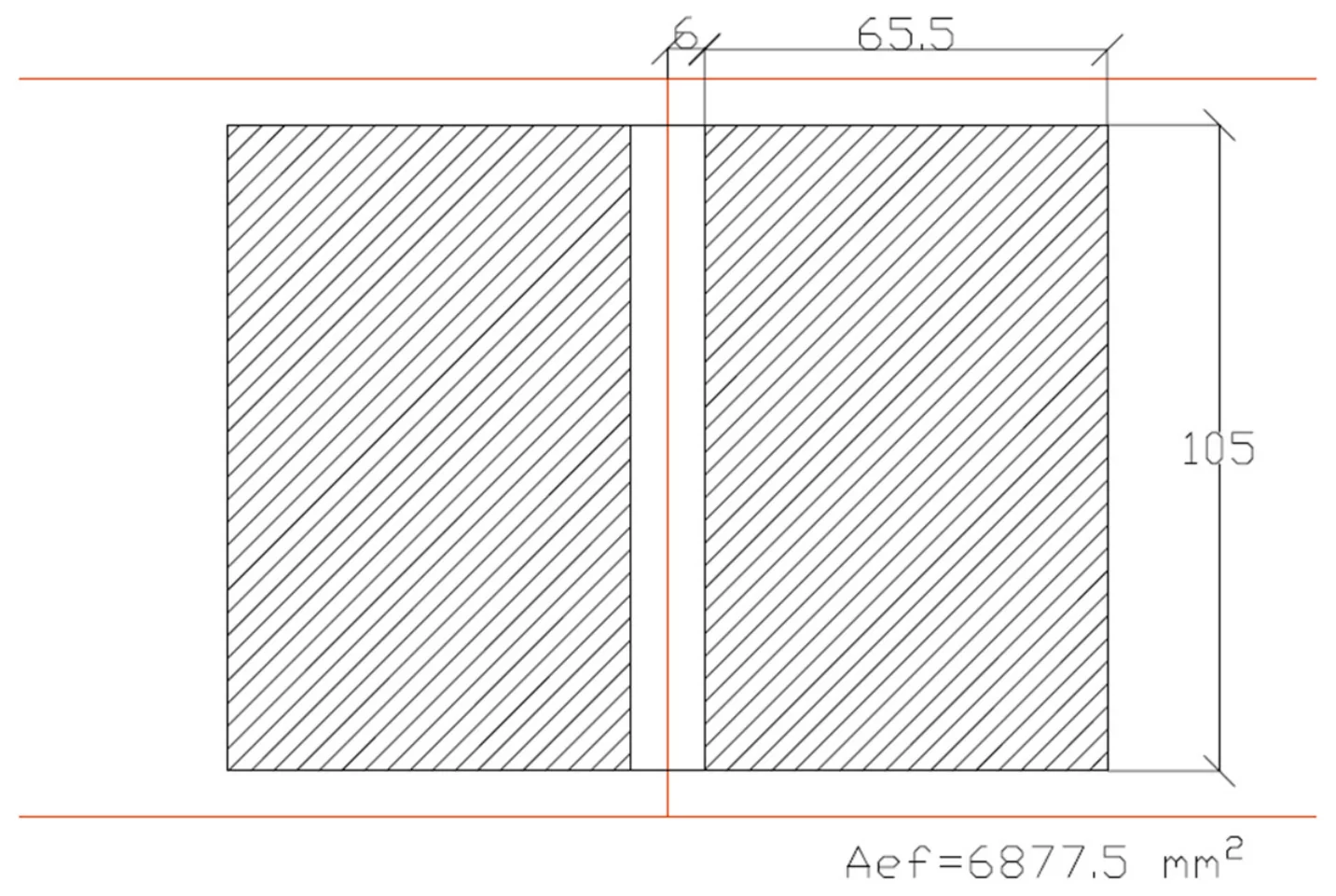
Effective anchorage area of one half of the GNA20-MIT plate used in the design verification. Edge and end reductions required by the connector model are excluded from the gross plate area; the resulting effective area is Aef = 6877.5 mm^2^ and is used to calculate anchorage stresses from axial force and bending moment (authors’ own drawing).

The effective area of the nail plate is shown in Figure 6.

$$A_{ef}=\left(\frac{143\;mm}{2}-6\;mm\right)\cdot 105\;mm=6877.5\;mm^{2}$$

$$F_{A,Ed}=\sqrt{{\left(\frac{F_{Ed}\cos\beta }{2}-\frac{3\left|M_{Ed}\right|}{2h}\right)}^{2}+{\left(F_{Ed}\sin\beta \right)}^{2}\;}$$

$$F_{A,Ed}=\sqrt{{\left(-\frac{3\left|0.54\right|}{2\cdot 0.12\;}\right)}^{2}}=6.75\;kN$$

$$M_{A_{Ed}}=\frac{M_{Ed}}{2}=\frac{0.54}{2}=0.27\;kNm$$

Determination of anchorage stress caused by axial force:

$$\tau_{F,d}=\frac{F_{A,Ed}}{A_{ef}}$$

$$\tau_{F,d}=\frac{6750}{6877.5}=0.9815\;\frac{N}{mm^{2}}$$

Calculation of the plate section modulus:

$$d=\sqrt{{\left(\frac{A_{ef}}{h_{ef}}\right)}^{2}+h_{ef}^{2}=\sqrt{{\left(\frac{6877.5}{65.5}\right)}^{2}+{\left(65.5\right)}^{2}}=123.76\;mm}$$

$$W_{p}=\frac{A_{ef}d}{4}=\frac{6877.5\cdot 123.76}{4}=212789.85\;mm^{3}$$

Determination of the anchorage stress caused by the bending moment:

$$\tau_{M,d}=\frac{M_{A,Ed}}{W_{p}}=\frac{270000}{212789.85}=1.27\;\frac{N}{mm^{2}}$$

Verification of the plate anchorage condition:

$${\left(\frac{\tau_{F,d}}{k_{mod}\frac{f_{a,\alpha ,\beta ,k}}{\gamma_{M}}}\right)}^{2}+{\left(\frac{\tau_{M,d}}{k_{mod}\frac{f_{a,0,0,k}}{\gamma_{M}}}\right)}^{2}\leq 1$$

$${{\left(\frac{0.9815}{0.9\cdot \frac{2.83}{1.3}}\right)}^{2}+\left(\frac{1.27}{0.9\cdot \frac{2.83}{1.3}}\right)}^{2}={{\left(\frac{0.9815}{1.9592}\right)}^{2}+\left(\frac{1.27}{1.9592}\right)}^{2}=0.81\leq 1$$

The load-bearing capacity condition of the plate due to anchorage has been met.

#### 3.3.2. Plate Capacity

Forces acting in combination:

$$F_{M,Ed}=\frac{2M_{Ed}}{l}=\frac{2\cdot 0.27}{0.105}=5143\;N$$

$$F_{x,Ed}=F_{Ed}\cos\alpha \pm 2F_{M,Ed}\sin\gamma =6750\cdot cos(0{}^{\circ})+2\cdot 5143\cdot sin(90{}^{\circ})=17036\;N$$

$$F_{y,Ed}=F_{Ed}\sin\alpha \pm 2F_{M,Ed}\cos\gamma =6750\cdot sin(0{}^{\circ})+2\cdot 5143\cdot cos(90{}^{\circ})=0.0\;N$$

Determination of the characteristic load-bearing capacity of the plates in the x and y directions:

$$f_{n,0,k}=f_{t,0,k}=152\;\frac{N}{mm}$$

$$f_{n,90,k}=f_{c,90,k}=70\;\frac{N}{mm}$$

k=1

$$F_{x,Rk}=\max\left\{\begin{array}{c} |f_{n,0,k}l\cdot \sin(\gamma -\gamma_{0}\sin2\gamma )|\\ \left|f_{v,0,k}l\cdot \cos\gamma \right| \end{array}\right.=\max\left\{\begin{array}{c} |152\cdot 105\cdot \sin(90{}^{\circ}+0.3\sin2\cdot 90{}^{\circ})|\\ \left|61\cdot 105\cdot \cos90\right| \end{array}\right.\;=15960\;N$$

$$F_{y,Rk}=\max\left\{\begin{array}{c} \left|f_{n,90,k}l\cdot \cos\gamma \right|\\ {|kf}_{v,90,k}l\cdot \sin\gamma | \end{array}\right.=\max\left\{\begin{array}{c} \left|70\cdot 105\cdot \cos90\right|\\ 42\cdot 105\cdot \sin90| \end{array}=4410\;N\right.$$

Checking the load-bearing capacity of the plate:

$$\frac{F_{x,Ed}}{k_{mod}\frac{F_{x,Rk}}{\gamma_{M}}}=\frac{17036}{0.9\frac{15960}{1.3}}=1.54\leq 1.0$$

The load-bearing capacity of the plate was exceeded by 54%.

The calculated result should be interpreted as a limit-state assessment within the adopted design model rather than as a direct prediction of specimen failure. In nail plate connections, stress distribution depends on the effective anchorage area, grain direction, contact between timber elements, and local load bearing. Consequently, small differences in plate geometry can produce substantial differences between calculated connector utilization and measured failure load [22,23].

### 3.4. Comparison of Manual Calculations with the Pamir Program

In order to compare the calculation results, an analysis of the internal forces and the load-bearing capacity of the wooden element and the plate was performed using the specialized Pamir program for designing roof structures connected with nail plates.

#### 3.4.1. Computational Model in Pamir

**Figure 7:**
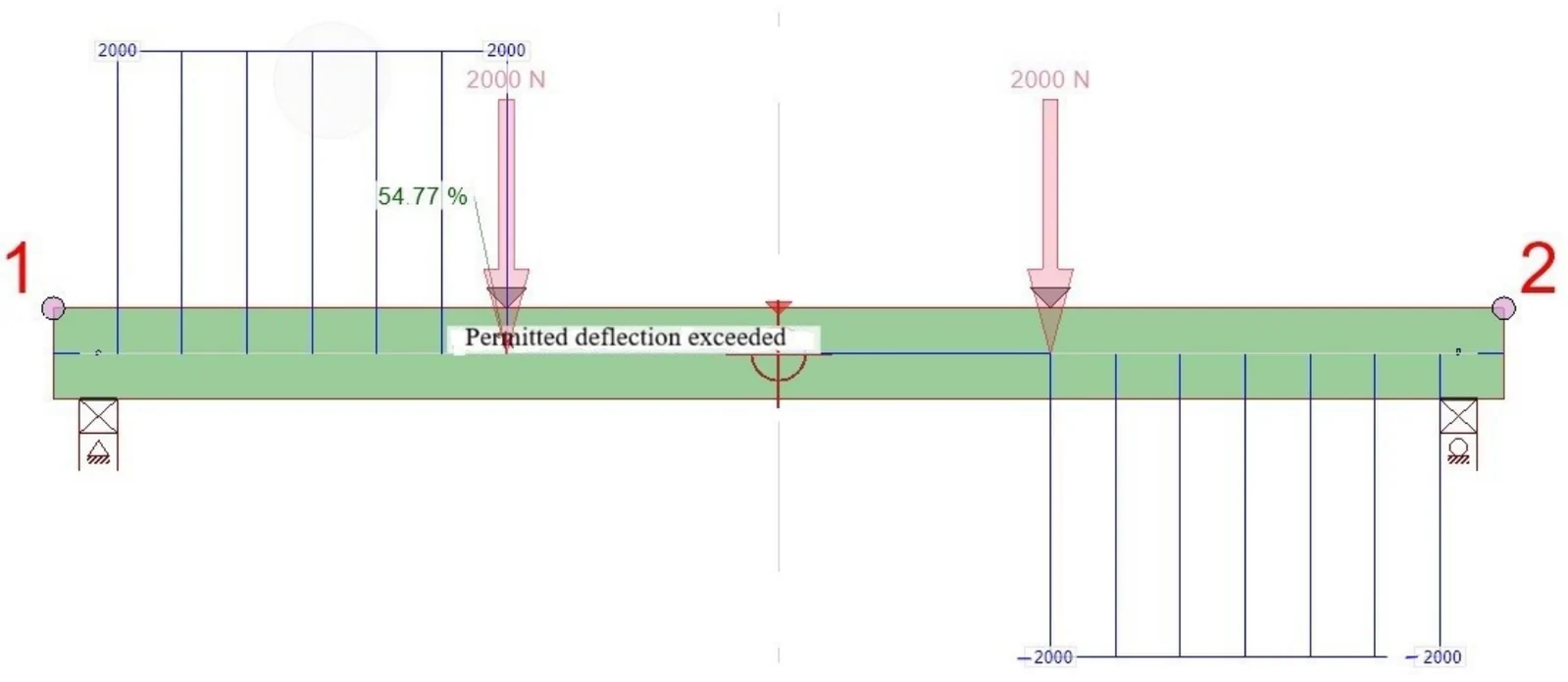
Pamir shear force output for the modeled specimen under two 2 kN point loads. The diagram reproduces the ±2 kN shear levels obtained from the manual solution and identifies the connector zone at midspan; the displayed utilization/deflection message relates to the adopted serviceability check (software output, authors’ own model).

The shear force distribution obtained from the Pamir model is presented in Figure 7.

**Figure 8:**
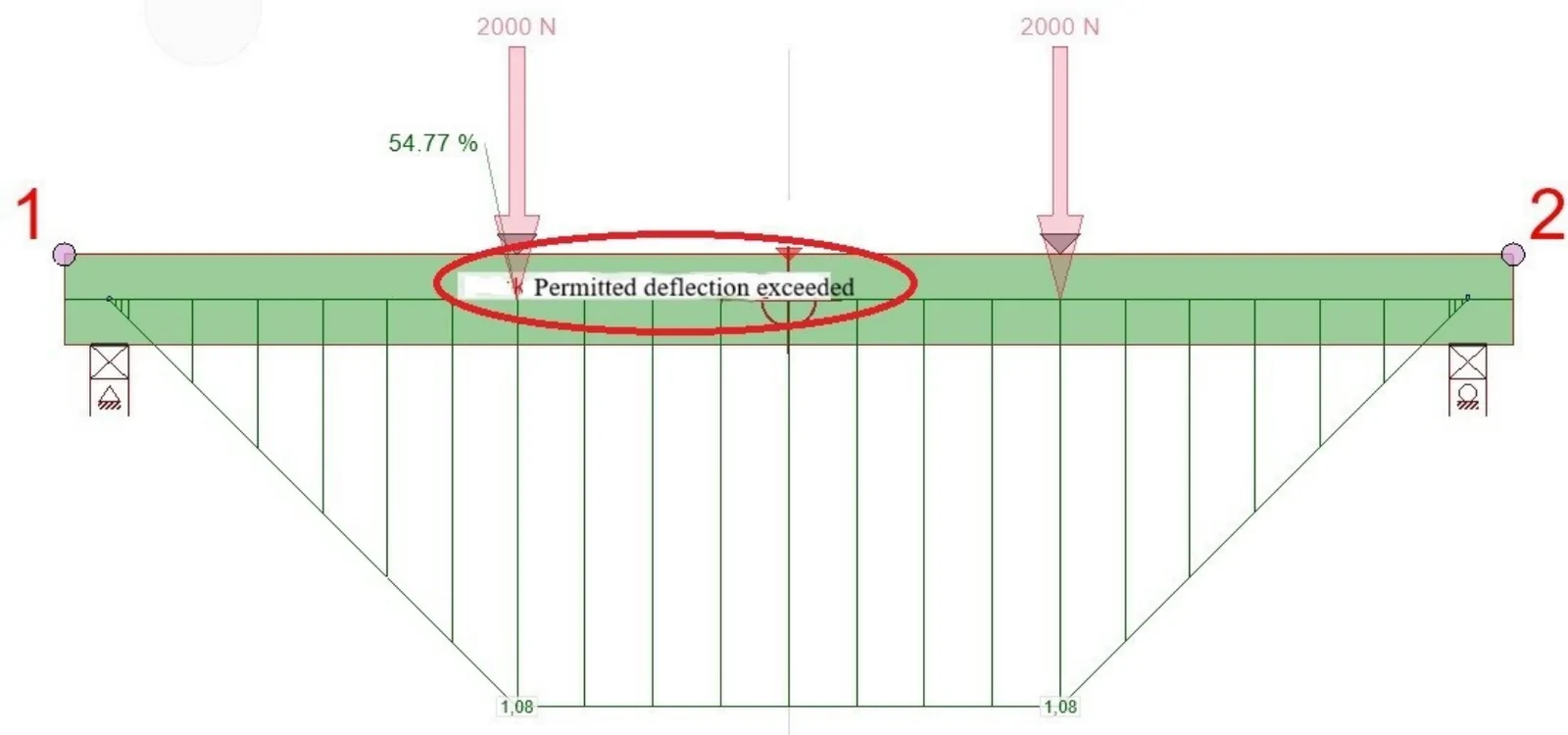
Pamir bending moment output for the same loading case. The triangular outer span diagrams and constant central moment agree with the analytical model, while the midspan indication marks the region in which joint flexibility most strongly affects global deflection (software output, authors’ own model).

The bending moment diagram in Figure 8 confirms agreement between the Pamir model and the analytical internal force solution.

#### 3.4.2. Load-Bearing Capacity of the Timber Cross-Section in Pamir

Analysis of the element’s bending and shear capacity (Table 5) indicates that the cross-section’s bending capacity according to the Pamir program was 54.8%. The calculated cross-section stress was 57.5%. The difference in results may be due to the additional bending efficiency factor assumed in the program. The cross-section’s shear capacity was 30%, which is exactly the same as the previously determined value.

The load-bearing capacity of the nail plate due to anchoring is lower by 1% in the Pamir program (Table 6). A detailed calculation report allows us to note the difference in the calculated plate strength index. The discrepancy results from the fact that we used a formula in the calculations that the standard defines as a conservative approximation of this index. The Pamir program is able to determine this index more precisely.

The load-bearing capacity of the plate due to linear rupture in the Pamir program is 15% higher. The difference results from the fact that the program does not use the coefficient to determine the calculated load-bearing capacity of the plate $k_{mod}$.

### 3.5. Physical Properties

Density is one of the key parameters in the practical use of wood. This is because the basic physical, mechanical, and technological properties of wood are directly related to it. A summary of the densities of individual samples based on the established testing methodology is presented in Table 7.

#### 3.5.1. Wood Density

The above data (Table 7) show that the sample density value ranges from 406.54 kg/m^3^ (sample A5) to 556.38 kg/m^3^ (sample M13). The average value was 462.80 kg/m^3^. The average sample density is therefore slightly higher than 420 kg/m3 C24 class wood, as assumed by the PN-EN 338 standard. The tested material has an average density 29.1 kg/m^3^ higher than the reference material.

The relative variability of both groups is small (CV ≈ 8%), indicating a fairly homogeneous material. The width of the 95% confidence interval is much larger for the reference material due to the small sample size (n = 5). The interquartile range is almost identical in both groups (~57 kg/m^3^), indicating a similar dispersion of the middle 50% of results.

The mean density exceeded the reference mean value for C24 timber, which may have improved resistance to local crushing and tooth anchorage. Density, moisture content, and local anatomical defects are among the principal timber variables affecting MPC joint stiffness and load-bearing capacity [6,22]. Nevertheless, the capacity of the connected specimens was governed by both timber properties and the retention of tooth anchorage in the joint zone.

#### 3.5.2. Moisture Content

The moisture content statistics for both specimen groups are presented in Table 8.

Each interval between Tests I and II and between Tests II and III included 6 days of laboratory drying after the 24 h immersion stage. The measured moisture contents therefore document that the specimens did not remain at a single hygroscopic state, but no inferential comparison between the reference and MPC moisture content distributions is used to support the mechanical conclusions.

The moisture content range is consistent with repeated changes in hygroscopic state during the conditioning sequence. Such changes can cause swelling and shrinkage and may alter tooth–wood contact [10]; however, the present global deflection measurements do not separately quantify local plate slip, tooth withdrawal, or timber bending.

### 3.6. Flexural Modulus

The apparent modulus of elasticity obtained in four-point bending is summarized for the continuous solid wood references in Table 9 and for the MPC specimens in Table 10. Calculations used the 1000–4000 N deformation range, corresponding to approximately 30% of ultimate load and to the linear portion of the load–deflection response.

**Figure 9:**
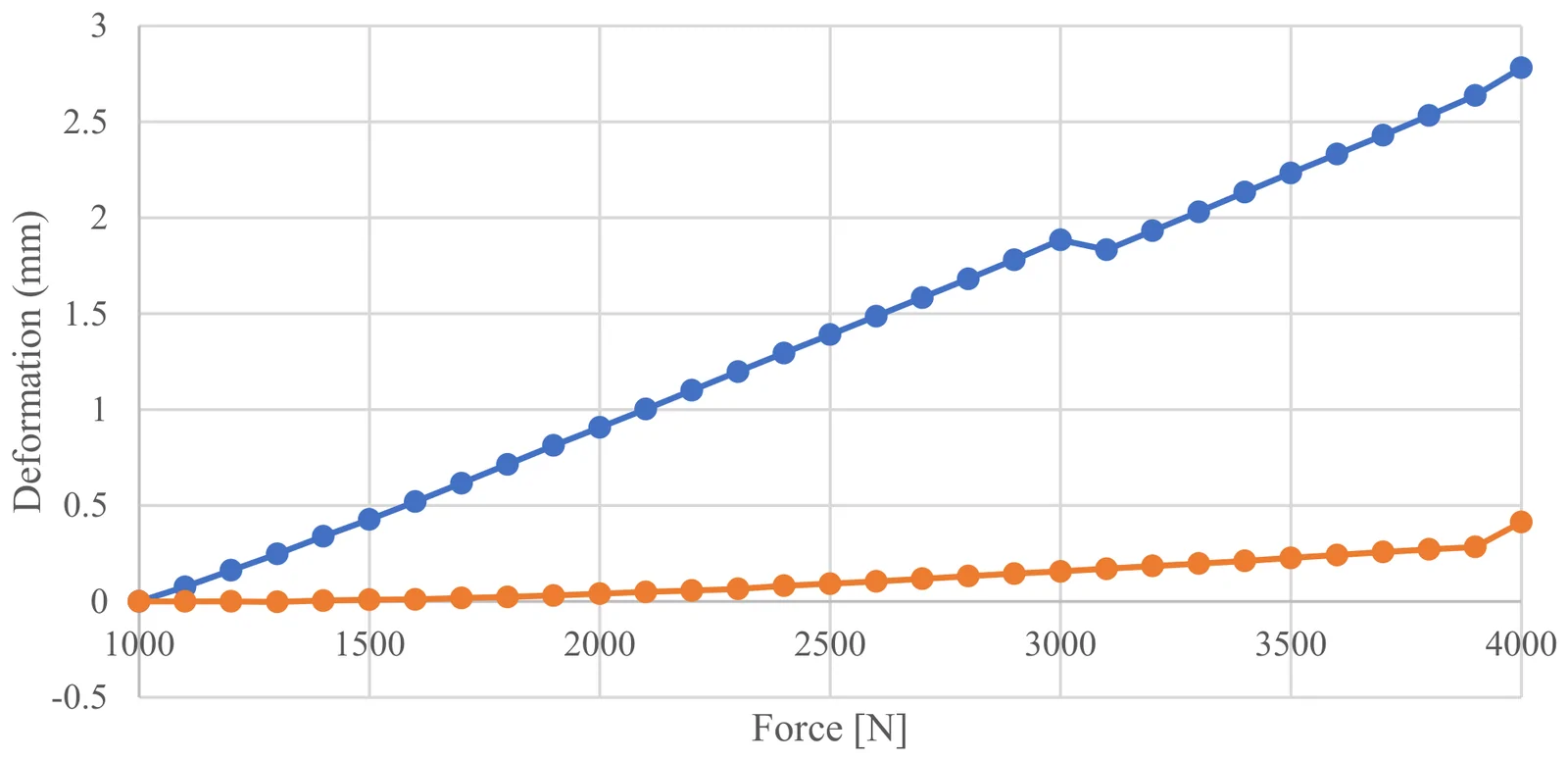
Mean unloading response in the 1000–4000 N evaluation range. MPC specimens exhibited substantially greater global deformation than continuous solid wood references. Because the instrumentation recorded global deflection, the plot is consistent with—but does not separately quantify—the contribution of joint slip and rotation.

The average deformation history of the samples during unloading is shown in Figure 9. Significant deformations during loading are indicated for the samples with nodes based on the nail plate connection.

An initial seating/preload step was excluded from the Test I modulus calculation because several MPC specimens exhibited nonrepresentative deformation associated with initial plate seating and closure of the butt joint gap. This observation also shows why repeated loading can contribute to later stiffness values independently of moisture conditioning.

The repeated apparent modulus values of the continuous reference series fluctuate markedly among Tests I–III (Table 9). Because n = 5 and the setup used a global dial gauge deflection measurement, these values are treated as descriptive only and are not used to infer a moisture-induced material response; repositioning, seating, moisture correction, and measurement sensitivity are possible contributors to the observed instability.

For the 15 MPC specimens, the mean apparent modulus decreased from 1.39 GPa in Test I to 1.33 GPa in Test II and 1.22 GPa in Test III, corresponding to an observed mean reduction of approximately 0.17 GPa (12%). The Friedman test did not show a statistically significant stage effect (χ^2^(2) = 4.13, *p* = 0.127; Kendall’s W = 0.138). Pairwise Wilcoxon tests were likewise non-significant (I–II *p* = 0.426; I–III *p* = 0.107; II–III *p* = 0.222). Accordingly, the 12% change is reported as an observed trend rather than as statistically confirmed degradation.

The direction of the observed mean change is consistent with mechanisms reported for MPC joints, including increased slip and relaxation of tooth–wood contact during moisture changes [10,11]. In the present experiment, however, local plate slip and tooth withdrawal were not measured independently, and no unexposed MPC control group was available; repeated loading and initial plate seating therefore remain plausible co-contributors.

**Figure 10:**
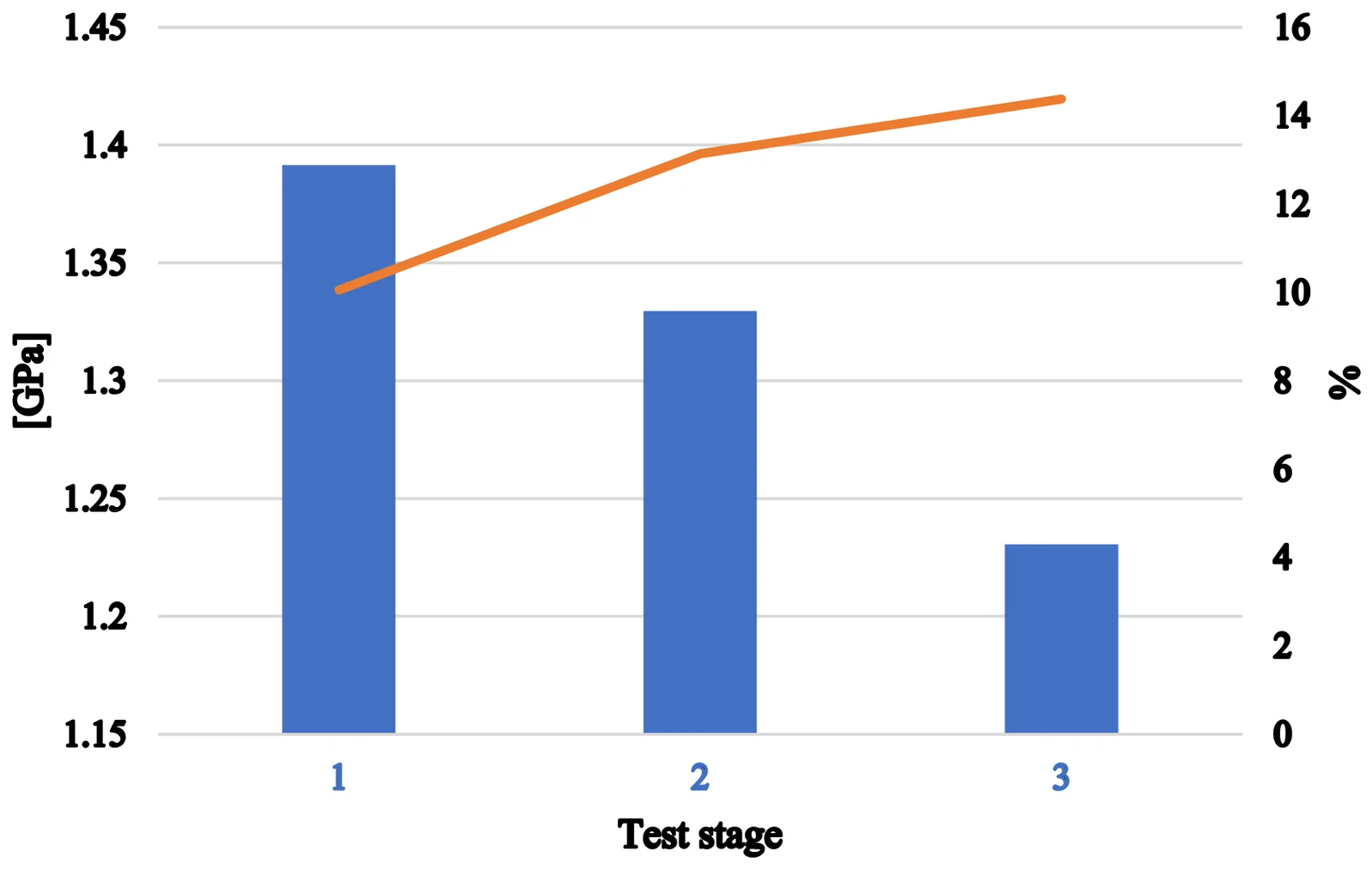
Mean apparent bending modulus of the MPC specimens across the three test stages, together with mean moisture content. The mean modulus changes from 1.39 to 1.22 GPa; the figure presents a descriptive association only and does not establish moisture cycling as the sole cause of the stiffness change.

Figure 10 compares the mean moisture content of the connected-specimen batch with the corresponding mean apparent modulus of elasticity at each test stage.

The descriptive trend is relevant to serviceability because additional connection flexibility can increase deformation in semi-rigid timber systems [6,24]. The magnitude measured here should not be used directly as a design reduction factor without an unexposed MPC control group, larger samples, and local joint displacement measurements.

### 3.7. Bending Strength, Ultimate Load, and Failure Mechanisms

Bending strength and ultimate load are summarized in Table 11 for the continuous solid wood references and in Table 12 for the MPC specimens.

Analysis of the reference material samples indicates strength values ranging from 23.87 MPa to 52.40 MPa, with an average of 34.14 MPa. The median result was 31.22 MPa.

The highest strength was recorded for sample A1 (52.40 MPa), while the lowest was for sample A2 (23.87 MPa). The range of results was 28.53 MPa, indicating a significant difference between extreme values. The calculated standard deviation of 11.11 MPa and the coefficient of variation of 32.55% indicate relatively large variability among the tested samples. For the ultimate strength, values ranged from 9123 N to 18,215 N, with a mean of 12,809 N. The median was 12,374 N, and the coefficient of variation was 27.03%, which also indicates noticeable variability in the results. Statistical analysis shows that the tested material is characterized by a moderately high average strength, but the results of individual samples are quite variable. The particularly high value obtained for sample A1 increases the arithmetic mean.

The final destructive test included all 15 MPC specimens. Their mean apparent bending strength was 16.92 MPa (median 17.40 MPa), with values ranging from 3.66 MPa for M7 to 22.60 MPa for M13. The mean ultimate load was 7100 N (median 7534 N), with a range from 1723 N to 8886 N. These values characterize the connected element tested in the present geometry and should not be interpreted as an intrinsic timber bending strength property.

A two-sided Grubbs test identified M7 as statistically unusual in the apparent strength series (G = 3.10, Gcrit = 2.55 at α = 0.05). Nevertheless, M7 failed prematurely during the third stiffness stage and may represent a genuine connection-level failure; it is therefore retained in the primary analysis.

With all 15 MPC specimens retained, the primary mean apparent bending strength is 16.92 MPa. Excluding M7 increases the mean to 17.87 MPa and is reported only as a sensitivity analysis. No C14 or other timber strength class assignment is made for MPC specimens, because their reported bending strength is an apparent property of the connected assembly rather than an intrinsic property of the timber.

**Figure 11:**
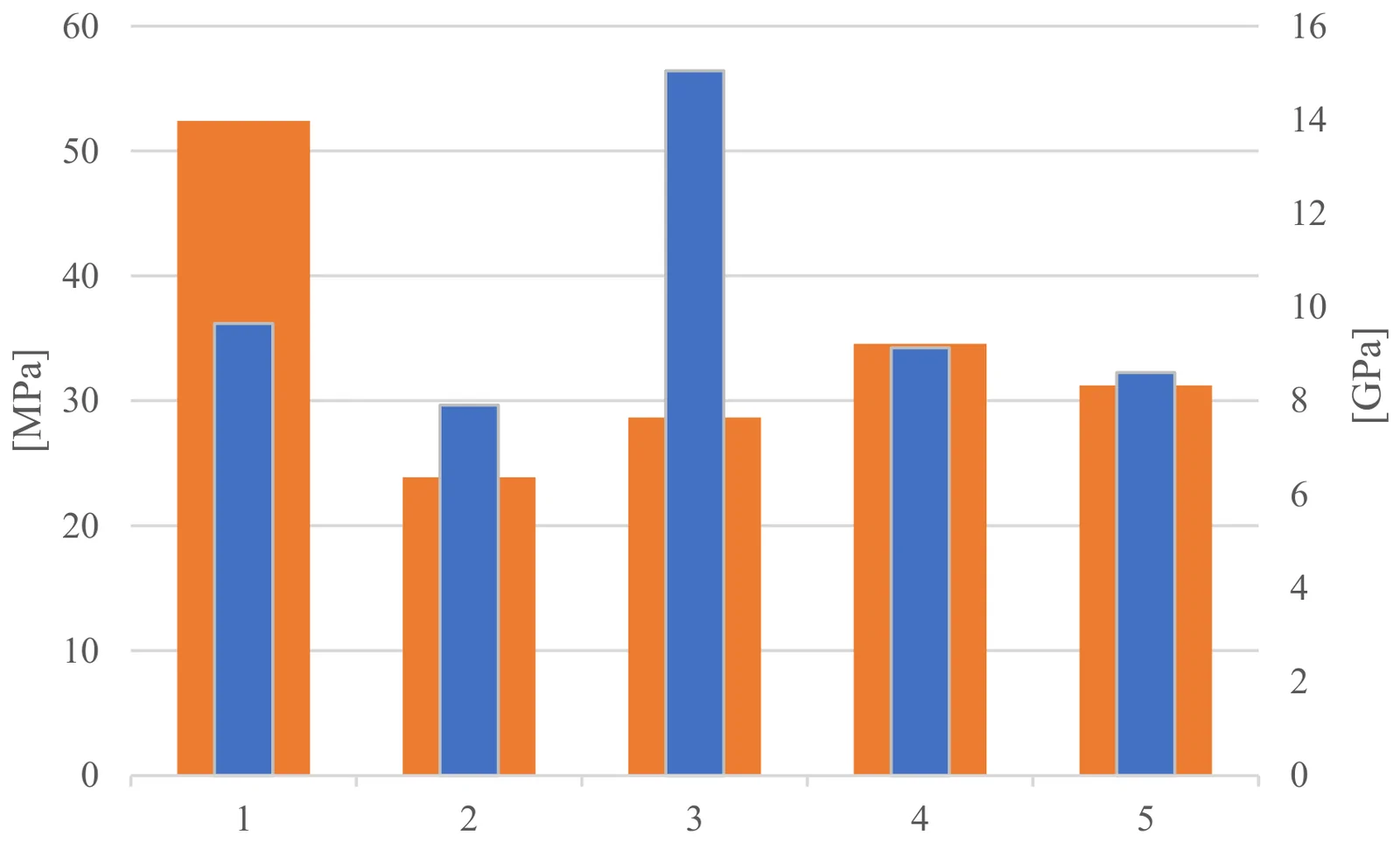
Specimen-by-specimen comparison of bending strength (MOR, orange; left axis) and mean modulus of elasticity (MOE, blue; right axis) for the five continuous solid wood references. The plot illustrates the large strength scatter associated with anatomical defects, including the exceptionally high result for A1 and the knot-controlled failure of A2.

**Figure 12:**
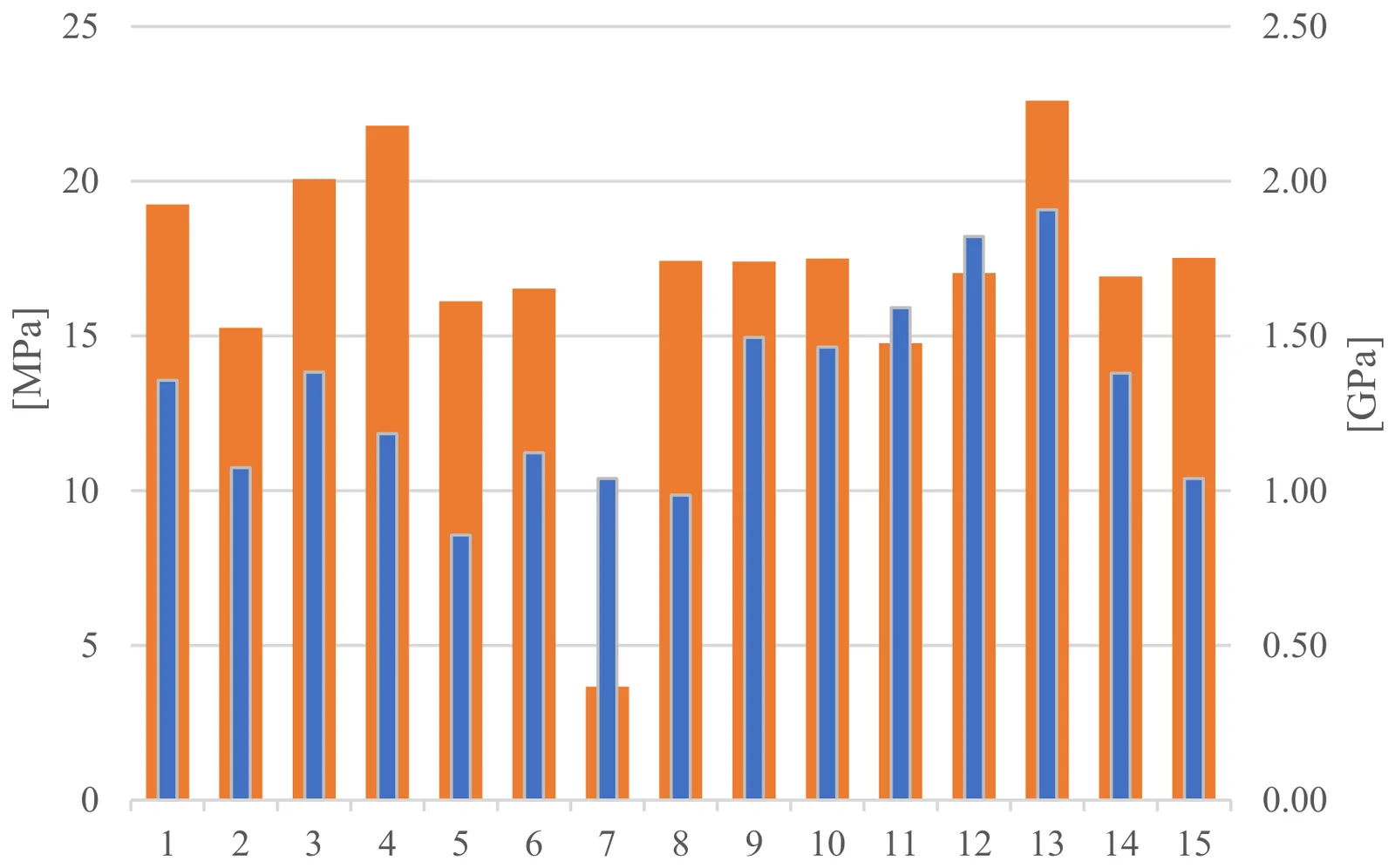
Specimen-by-specimen comparison of apparent bending strength of the connected element (MOR, orange; left axis) and mean apparent modulus of elasticity (MOE, blue; right axis) for the 15 MPC specimens. M7 is retained and is distinguished by premature connection damage during the third stiffness stage.

**Figure 13:**
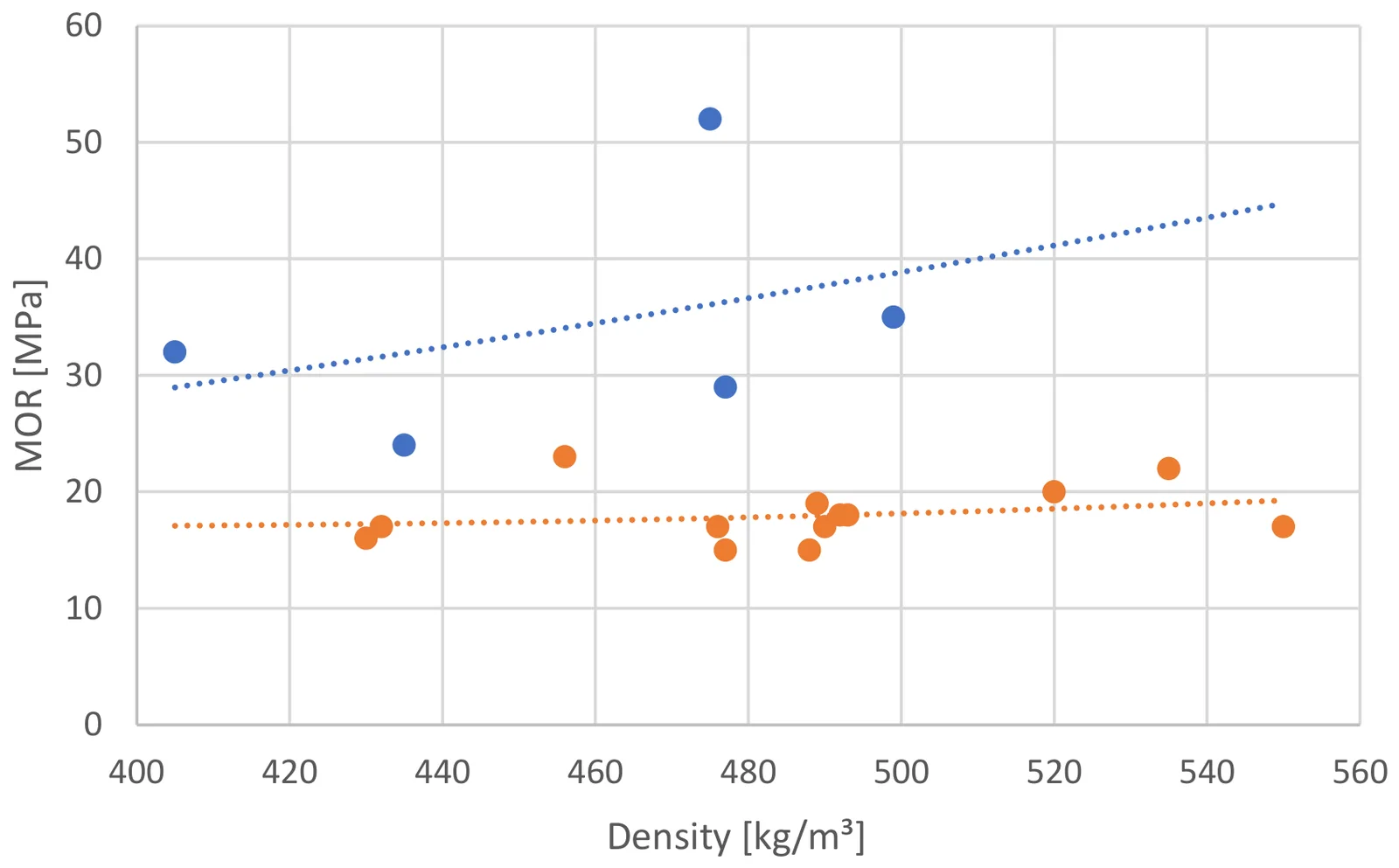
Relationship between wood density and bending response. Blue markers represent the bending strength of continuous reference specimens, and orange markers represent the apparent bending strength of MPC assemblies; dotted polynomial trend lines are shown separately for each group. The plot is descriptive, and the two groups should not be interpreted as mechanically equivalent configurations.

Figure 11 and Figure 12 compare specimen-level bending strength with the mean modulus of elasticity for the reference and connected groups, respectively. Figure 13 then relates bending strength to wood density.

## 4. Discussion

The continuous reference specimens and the MPC specimens represent fundamentally different structural configurations. In the reference series, final failure was governed by timber cross-section properties and local anatomical defects; in the MPC series, the observed failure was generally progressive and involved plate slip, local crushing, and partial tooth withdrawal [6,22]. Therefore, direct comparison of their final strengths characterizes the effect of introducing a central connected butt joint, not the isolated effect of moisture cycling.

**Figure 14:**
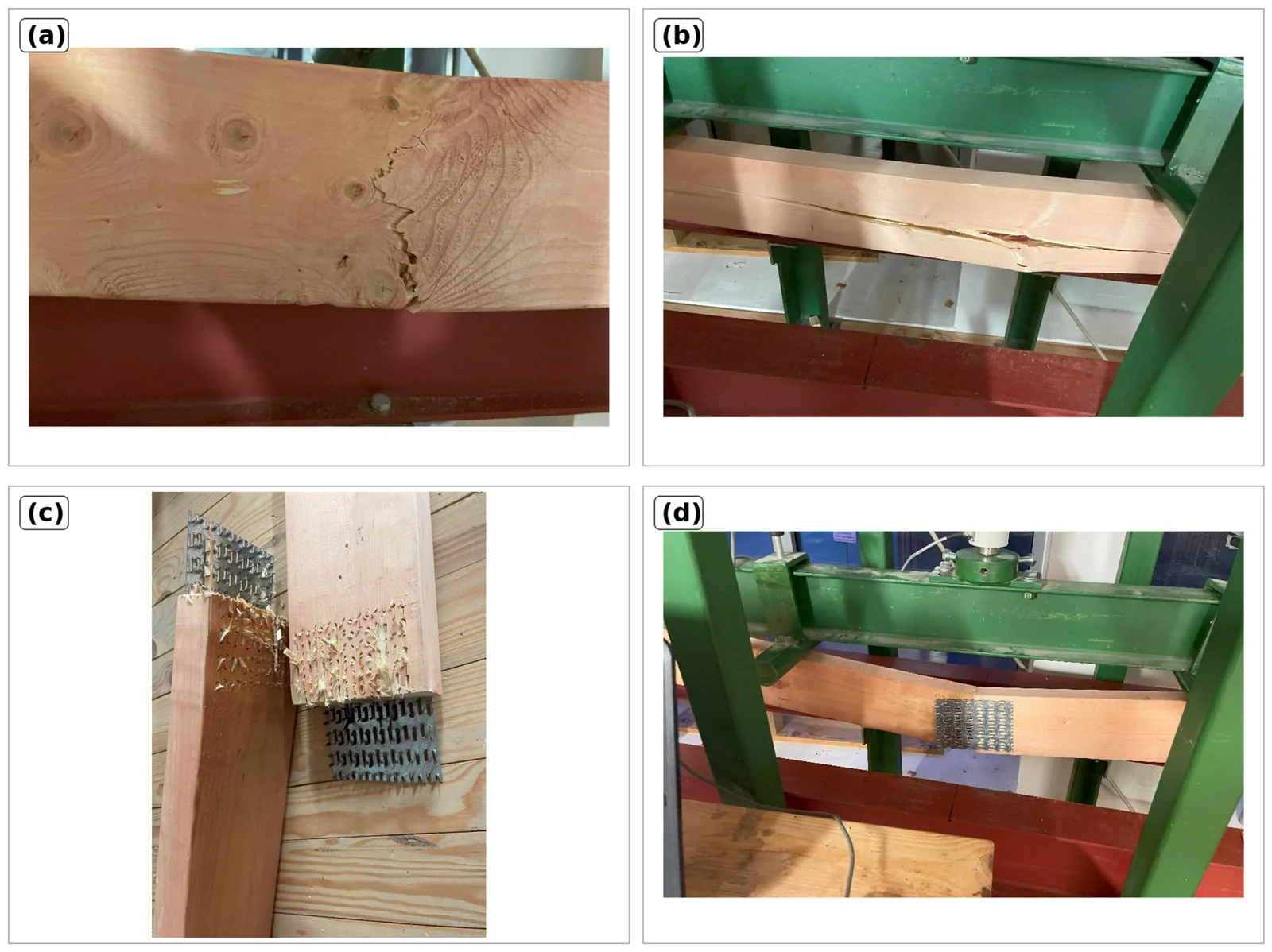
Representative failure mechanisms observed after four-point bending: (**a**) transverse crack through the knot zone in reference specimen A2, producing the lowest solid wood strength; (**b**) longitudinal splitting and abrupt timber fracture in reference specimen A1; (**c**) separated MPC specimen M7 showing progressive tooth withdrawal, local fiber crushing, and loss of effective anchorage on both members; (**d**) connected specimen in the test frame immediately after large joint rotation and partial plate pullout. Panels (**a**,**b**) illustrate brittle, timber-controlled failure, whereas panels (**c**,**d**) illustrate the more gradual, connection-controlled mechanism.

Representative timber-controlled and connection-controlled failure patterns are documented in Figure 14.

The primary strength analysis retains all 15 MPC specimens: the mean apparent bending strength of the connected assemblies was 16.92 MPa, compared with 34.14 MPa for the continuous reference specimens. The approximately 50% difference primarily reflects the central butt joint and connector-controlled load path and must not be described as moisture-induced strength degradation. M7 satisfies the Grubbs statistical outlier criterion, but it remains in the primary analysis because its premature failure may be mechanically meaningful; the 17.87 MPa mean obtained after excluding M7 is reported only as a sensitivity result.

The mean apparent MPC modulus decreased by approximately 12% from Test I to Test III, but the repeated-measures analysis was not statistically significant (*p* = 0.127). Moreover, the absence of an unexposed MPC control group means that moisture cycling cannot be separated from repeated loading, initial plate seating, and progressive joint slip. The tooth–wood relaxation mechanism is therefore a literature-supported interpretation consistent with the observed failure modes [10,11], not a mechanism directly demonstrated by local displacement, microscopy, or single-tooth pull-out testing in this study.

For descriptive engineering comparison, a stiffness retention ratio may be written as rE = Eapp,t/Eapp,I. Using the group means yields rE = 1.22/1.39 = 0.88 for Test III. This test-specific value is not proposed as a Eurocode 5 modification factor or design coefficient, because the stage effect was not statistically significant, and the experimental design did not isolate moisture as a single variable. Eurocode 5 service class provisions therefore remain the appropriate normative framework for design [4].

The present work also does not quantify cycle-by-cycle ultimate capacity degradation: ultimate load was measured only after Test III. Consequently, the data support comparison of stiffness trajectories and final failure response, but not a degradation law for ultimate resistance as a function of cycle number. A larger reference series and an unexposed MPC control series would be required for stronger inferential conclusions.

The practical implication is limited to serviceability awareness: changes in connection flexibility can influence deflection and internal force redistribution in semi-rigid timber systems [6,24,25,26,27,28,29,30,31,32,33,34]. The present results justify monitoring connection stiffness under variable environmental histories, but they do not establish a universal moisture reduction factor.

The Pamir calculations reproduce the order of magnitude of the internal forces and provide a useful design comparison for the tested geometry. The experimental data were not used to perform a formally validated model-updating procedure, and claims of quantified resource or environmental benefit have therefore been removed. Further work should combine unexposed MPC controls, larger reference groups, local displacement sensors or DIC, microscopy of the tooth–wood interface, single-tooth or plate pull-out tests, cycle-resolved destructive testing, moisture profiling near the interface, corrosion assessment of the Z275 coating, and coupled creep–humidity loading.

## 5. Conclusions

Across the 15 MPC specimens, the mean apparent modulus decreased from 1.39 GPa in Test I to 1.22 GPa in Test III (approximately 12%). This stage-to-stage trend was not statistically significant in the Friedman repeated-measures analysis (*p* = 0.127), and the absence of an unexposed MPC control group prevents attribution of the change exclusively to moisture cycling.

In the final destructive test, the primary analysis retaining all MPC specimens gave a mean apparent bending strength of 16.92 MPa, compared with 34.14 MPa for the continuous reference specimens. This difference reflects the fundamentally different structural configurations and is not evidence of a 50% moisture-induced strength loss. M7 is retained in the primary analysis; the 17.87 MPa mean after its exclusion is presented only as a sensitivity result, and no timber strength class is assigned to the MPC assemblies.

MPC failures were generally progressive and involved plate slip and partial spike withdrawal, indicating the importance of connection deformation for serviceability assessment. The Pamir model is used as a design comparison rather than as a validated model-updating result. Future studies should add unexposed MPC controls, larger reference samples, local joint displacement measurements, cycle-resolved ultimate tests, microscopic and pull-out characterization of the anchorage zone, interface moisture profiling, corrosion assessment, and coupled creep–humidity loading.

## Figures and Tables

**Table 1:** Characteristic and mean properties of C24 Norway spruce adopted for the study, on the basis of EN 338.

Property	Unit	Symbol	Value
Bending strength	MPa	*f_m,k_*	24
Tensile strength (along the grain)	MPa	*f_t,_*_0,*k*_	14
Compressive strength (along the grain)	MPa	*f_c,_*_0,*k*_	21
Shear strength	MPa	*f_v,k_*	4.0
Compressive strength (perpendicular to the grain)	MPa	*f_c,90,k_*	2.5
Tensile strength (perpendicular to the grain)	MPa	*f_t,_*_90,*k*_	0.4
Modulus of elasticity (medium)	MPa	*E_0,mean_*	11,000
Modulus of elasticity (5% fraction)	MPa	*E*_0.05_	7400
Modulus of elasticity transverse	MPa	*E*_90,*mean*_	370
Shear modulus	MPa	*G_mean_*	690
Density characteristic	kg/m^3^	*ρ_k_*	350
Density mean	kg/m^3^	*ρ_mean_*	420
Reference moisture content	%	*w_ref_*	approx. 12
Conductivity thermal		*λ*	0.13

**Table 2:** Specimen groups, identifiers, geometry, and function in the experimental program.

Series and Identifiers	n	Specimen Geometry and Connection	Function in the Study
Reference series A1–A5	5	Continuous C24 spruce member, 1920 × 45 × 120 mm; no mechanical connector or midspan discontinuity.	Baseline response of solid timber: density, moisture content, apparent bending modulus, bending strength, ultimate load, and timber-controlled failure mode.
MPC series M1–M15	15	Two C24 spruce members joined at midspan by a butt joint; one GNA20-MIT plate (105 × 143 × 1.0 mm) pressed on each face at approximately 18 t.	Assessment of moisture-sensitive joint stiffness and strength, comparison with the computational model, and identification of slip, local crushing, and tooth withdrawal failure.

**Table 3:** Symbols and resistance parameters used for nail plate anchorage and in-plane plate-capacity calculations.

Symbol	Description
*f_a,_*_90_ *_,_*_90_	anchorage load capacity per unit area for α = 90°, and β = 90°
*f_t,_*_0_	tensile load capacity per unit width of the plate α = 0°
*f_c,_*_0_	compression load capacity per unit width of the plate α = 0°
*f_v,_*_0_	shear capacity per unit of plate dimension in the x direction
*f_t,_*_90_	tensile load capacity per unit width of the plate α = 90°
*f_c,_*_90_	compression load capacity per unit width of the plate α = 90°
*f_v,_*_90_	shear capacity per unit of plate dimension in the y direction
*k_1_*, *k_2_*, *α*_0_	constants

Where: x direction—main direction of the plate, y direction—direction perpendicular to the main direction of the plate, α—angle between the x direction and the force (stretching: 0°≤γ<90°,pressure 90°≤γ<180°), β—angle between the fiber direction (2) and the force, γ—angle between the x direction and the contact line, l—board dimension measured along the line of contact of the elements.

**Table 4:** Declared material, anchorage, in-plane resistance, stiffness, ductility, and durability parameters of the GNA20-MIT nail plate [14].

Essential Characteristics of Nail Plates		
Essential Characteristic	Declared Property	Harmonized Technical Specification
Steel	S250GD + Z275 NAC/MAC/MBC	EN 10143:2006 and EN 10346:2009
Thickness	1.0 mm	EN 14545:2008
Characteristic anchorage strength of plate 1: Solid and glued laminated timber with characteristic density ρ k = 350 kg/m^3^	fa,0.0 = 2.83 N/mm^2^ fa,90.90 = 1.63 N/mm^2^ k1 = −0.013k2 = 0.0004 α 0 = 29.0°	
Characteristic tensile, compressive, and shear strength of the plate	ft.0 = 152 N/mm; ft,90 = 83 N/mm fc,0 = 89 N/mm; fc,90 = 70 N/mmfv,0 = 61 N/mm; fv,90 = 42 N/mm ν 0 = −0.30; ky = 0.87	
Modulus of elasticity at average wood density ρ k = 350 kg/m^3^	kser,mean = 13.1 N/mm^3^	
Tooth ductility	Fulfilled	
Minimum timber thickness	35 mm	
Durability and corrosion protection	Z275 galvanized coating	
Service class	2	EN 1995-1-1

Note: tables were reproduced from submitted figures; values were saved as text to preserve decimal points, and units are as shown in the image.

**Table 5:** Pamir calculation results for the most unfavorable load combination, including bending and shear utilization of the C24 timber member.

Parameter							Value			
Combination loads							10,003			
kh							1.05			
kmod							0.9			
γM							1.3			
Factor out-of-plane buckling							1			
Factor performance bending							1.05			
kv							1			
kcr							0.67			
Force axial N							0			
Force shear N							2000			
Axial CSI							0.0%			
CSI Shear							30.0%			
**Items/Result calculations**										
Element/nodes	Distance [mm]	Distance [%]	Height [mm]	Class	Length buckling [mm]	Twisting—length [mm]	Moment [kNm]	Bending CSI [%]	Twisting [%]	Equ./Max CSI [%]
1–2	600	31	120	C24	1920x	1920	1.08	54.8	54.8	6.11/54.8
—	85	4	—	—	0	—	0.05	2.6	2.6	6.13/30.0

**Table 6:** Pamir verification of GNA20-MIT connector anchorage and linear plate rupture for the modeled joint.

Parameter				Value													
Type connector				Nail plate GNA20 105 × 143													
Truss lifting resistance				1192 N													
**Verification anchorages**																	
KO	From-To Element	Aef [mm^2^]	WP [cm^2^]	Force [N]	Angle [°]	Moment [kNm]	Reduced values—force [N]	Reduced values—angle [°]	Reduced values—moment [kNm]	Fa,α,β [N/mm^2^]	Fa,0,0 [N/mm^2^]	α	β	Modified [N/mm^2^]	N/mm^2^	Hand [mm^2^]	CSI [%]
10003	s1-1	6878	225.30	0	90	0.54	6750	180	0.27	2.83	2.83	0	0	1.96	1.96	528	**80**
10003	s1-2	6878	225.30	0	90	−0.54	6750	0	−0.27	2.83	2.83	0	0	1.96	1.96	528	**80**
**Verification ruptures—linear**																	
KO	Involved points	Left [mm]	Force [N]	Angle [°]	Moment [kNm]	Fx,Ed [N/mm]	Fy,Ed [N/mm]	Fx,Rd [N/mm]	Fy,Rd [N/mm]	γ [°]	FhEd [N/mm]	FhRd [N/mm]	Hand [mm]	CSI [%]			
10003	0 -> 1	105	6750	0	0.27	162.24	0	116.92	32,31	90	5.68	27.77	21	139			

**Table 7:** Descriptive statistics of wood density at the 12% reference moisture content for continuous reference specimens and MPC specimens.

Statistics	Reference Specimens (A)	Test Specimens (M)
Number samples (n)	5	15
Average [kg/m^3^]	461.42	490.52
Median [kg/m^3^]	475.89	489.44
Minimum [kg/m^3^]	406.54	427.97
Maximum [kg/m^3^]	498.66	556.38
Range [kg/m^3^]	92.12	128.41
Variance [kg^2^/m^6^]	1565.96	1557.77
Standard deviation (s) [kg/m^3^]	39.57	39.47
Coefficient of variation (CV) [%]	8.58	8.05
Standard error of the mean (SEM) [kg/m^3^]	17.7	10.19
First quartile (Q1) [kg/m^3^]	434.28	454.39
Third quartile (Q3) [kg/m^3^]	491.71	511.62
Interquartile range (IQR) [kg/m^3^]	57.43	57.23
95% confidence interval of the mean [kg/m^3^]	412.26–510.57	468.66–512.39

**Table 8:** Descriptive statistics of measured moisture content for continuous reference specimens and MPC specimens across the test sequence.

Statistics	Reference Specimens (A)	Test Specimens (M)
Number samples (n)	5	15
Mean [%]	14.16	11.95
Median [%]	13.78	11.93
Minimum [%]	11.48	9.01
Maximum [%]	16.01	15.51
Range [%]	4.53	6.5
Variance [%^2^]	3.98	3.59
Standard deviation (s) [%]	2	1.89
Coefficient of variation (CV) [%]	14.08	15.85
Standard error of the mean (SEM) [%]	0.89	0.49
First quartile (Q1) [%]	12.98	10.53
Third quartile (Q3) [%]	15.19	13.07
Interquartile range (IQR) [%]	2.21	2.54
95% confidence interval of the mean [%]	11.68–16.64	10.90–12.99

**Table 9:** Apparent modulus of elasticity in four-point bending for each continuous solid wood reference specimen, corrected to 12% moisture content.

Sample No.	Test I	Test II	Test III
	GPa		
A1	10.37	15.11	3.47
A2	3.71	5.53	14.48
A3	9.11	13.84	22.18
A4	9.38	12,13	5.88
A5	2.77	4.86	18.17
Min	2.77	4.86	3.47
Mean	7.07	10.29	12.84
Max	10.37	15.11	22.18
Standard deviation	3.17	4.28	7.14

**Table 10:** Apparent modulus of elasticity in four-point bending for each MPC specimen, corrected to 12% moisture content.

Sample No.	Test I	Test II	Test III
	GPa		
M1	0.99	1.56	1.52
M2	1.14	1.16	0.92
M3	1.50	1.37	1.28
M4	1.15	1.13	1.27
M5	0.95	0.87	0.75
M6	1.41	1.08	0.88
M7	1.41	0.84	0.87
M8	1.40	0.70	0.85
M9	1.47	1.89	1.12
M10	1.67	1.49	1.23
M11	1.59	1.92	1.26
M12	1.74	1.41	2.32
M13	1.89	1.87	1.96
M14	1.43	1.71	1.00
M15	1.13	0.95	1.04
Min	0.95	0.70	0.75
Mean	1.39	1.33	1.22
Max	1.89	1.92	2.32
Standard deviation	0.27	0.39	0.42

**Table 11:** Descriptive statistics of bending strength and ultimate load for the continuous solid wood reference group at 12% reference moisture content.

Parameter	Strength [MPa]	Failure Load [N]
Number of samples (n)	5	5
Arithmetic mean	34.14	12,809
Median	31.22	12,374
Minimum	23.87	9123
Maximum	52.4	18,215
Range	28.53	9092
Standard deviation	11.11	3463
Variance [%]	123.35	27.03
Coefficient of variation (CV)	32.55%	5

**Table 12:** Descriptive statistics of final ultimate load and apparent bending strength for the MPC specimen group at 12% reference moisture content; all 15 specimens are retained in the primary analysis.

Parameter	Failure load [N]	Apparent Bending Strength of Connected Element [MPa]
Number of samples (n)	15	15
Arithmetic mean	7100.2	16.92
Median	7534	17.40
Minimum	1723	3.66
Maximum	8886	22.60
Range	7163	18.94

## Data Availability

The original contributions presented in this study are included in the article. Further inquiries can be directed to the corresponding author.
